# Androgens In Men Study (AIMS): protocol for meta-analyses of individual participant data investigating associations of androgens with health outcomes in men

**DOI:** 10.1136/bmjopen-2019-034777

**Published:** 2020-05-11

**Authors:** Bu Beng Yeap, Ross James Marriott, Robert J Adams, Leen Antonio, Christie M Ballantyne, Shalender Bhasin, Peggy M Cawthon, David John Couper, Adrian S Dobs, Leon Flicker, Magnus Karlsson, Sean A Martin, Alvin M Matsumoto, Dan Mellström, Paul E Norman, Claes Ohlsson, Eric S Orwoll, Terence W O'Neill, Molly M Shores, Thomas G Travison, Dirk Vanderschueren, Gary A Wittert, Frederick C W Wu, Kevin Murray

**Affiliations:** 1 Medical School, University of Western Australia, Perth, Western Australia, Australia; 2 Department of Endocrinology and Diabetes, Fiona Stanley Hospital, Perth, Western Australia, Australia; 3 School of Population and Global Health, University of Western Australia, Perth, Western Australia, Australia; 4 Adelaide Institute for Sleep Health, Flinders University, Bedford Park, South Australia, Australia; 5 Clinical and Experimental Endocrinology, KU Leuven, Leuven, Belgium; 6 Internal Medicine, Baylor College of Medicine, Houston, Texas, USA; 7 Harvard Medical School, Boston, Massachusetts, USA; 8 San Francisco Coordinating Center, California Pacific Medical Center Research Institute, San Francisco, California, USA; 9 Gillings School of Global Public Health, University of North Carolina at Chapel Hill, Chapel Hill, North Carolina, USA; 10 School of Medicine, Division of Endocrinology, Diabetes and Metabolism, Johns Hopkins University, Baltimore, Maryland, USA; 11 WA Centre for Health & Ageing, University of Western Australia, Perth, Western Australia, Australia; 12 Department of Clinical Sciences and Orthopedic Surgery, Lund University, Lund, Sweden; 13 Freemasons Foundation Centre for Men's Health, The University of Adelaide, Adelaide, South Australia, Australia; 14 Geriatric Research, Education and Clinical Center, VA Puget Sound Health Care System, Seattle, Washington, USA; 15 Department of Medicine, Division of Gerontology & Geriatric Medicine, University of Washington School of Medicine, Seattle, Washington, USA; 16 Centre for Bone and Arthritis Research at the Sahlgrenska Academy, Institute of Medicine, University of Gothenburg, Goteborg, Sweden; 17 Oregon Health & Science University, Portland, Oregon, USA; 18 Centre for Epidemiology Versus Arthritis, Faculty of Biology, Medicine and Health, The University of Manchester & NIHR Manchester Biomedical Research Centre, Manchester, UK; 19 Manchester University NHS Foundation Trust, Manchester Academic Health Science Centre, Manchester, UK; 20 VA Puget Sound Health Care System, Seattle, Washington, USA; 21 School of Medicine, Department of Psychiatry and Behavioral Sciences, University of Washington, Seattle, Washington, USA; 22 Institute for Aging Research, Hebrew SeniorLife, Beth Israel Deaconess Medical Center, Boston, Massachusetts, USA; 23 Department of Chronic Diseases, Metabolism and Ageing (CHROMETA), Laboratory of Clinical and Experimental Endocrinology, Katholieke Universiteit Leuven, Leuven, Flanders, Belgium; 24 Division of Diabetes, Endocrinology and Gastroenterology, The University of Manchester, Manchester, UK

**Keywords:** sex steroids & HRT, epidemiology, general endocrinology

## Abstract

**Introduction:**

This study aims to clarify the role(s) of endogenous sex hormones to influence health outcomes in men, specifically to define the associations of plasma testosterone with incidence of cardiovascular events, cancer, dementia and mortality risk, and to identify factors predicting testosterone concentrations. Data will be accrued from at least three Australian, two European and four North American population-based cohorts involving approximately 20 000 men.

**Methods and analysis:**

Eligible studies include prospective cohort studies with baseline testosterone concentrations measured using mass spectrometry and 5 years of follow-up data on incident cardiovascular events, mortality, cancer diagnoses or deaths, new-onset dementia or decline in cognitive function recorded. Data for men, who were not taking androgens or drugs suppressing testosterone production, metabolism or action; and had no prior orchidectomy, are eligible. Systematic literature searches were conducted from 14 June 2019 to 31 December 2019, with no date range set for searches. Aggregate level data will be sought where individual participant data (IPD) are not available. One-stage IPD random-effects meta-analyses will be performed, using linear mixed models, generalised linear mixed models and either stratified or frailty-augmented Cox regression models. Heterogeneity in estimates from different studies will be quantified and bias investigated using funnel plots. Effect size estimates will be presented in forest plots and non-negligible heterogeneity and bias investigated using subgroup or meta-regression analyses.

**Ethics and dissemination:**

Ethics approvals obtained for each of the participating cohorts state that participants have consented to have their data collected and used for research purposes. The Androgens In Men Study has been assessed as exempt from ethics review by the Human Ethics office at the University of Western Australia (file reference number RA/4/20/5014). Each of the component studies had obtained ethics approvals; please refer to respective component studies for details. Research findings will be disseminated to the scientific and broader community via the publication of four research articles, with each involving a separate set of IPD meta-analyses (articles will investigate different, distinct outcomes), at scientific conferences and meetings of relevant professional societies. Collaborating cohort studies will disseminate findings to study participants and local communities.

**PROSPERO registration number:**

CRD42019139668.

**Strengths and limitations of this study**

- The individual participant data (IPD) meta-analyses are likely to have higher statistical power and provide greater scope to control for important confounders and risk factors than previous meta-analyses on this topic.
- Investigators from nine large prospective cohort studies (each with n>1000 participants) have agreed to collaborate with additional studies to be identified from systematic review.
- Harmonisation will be required for some variables (eg, physical activity, alcohol consumption) that are recorded differently among the component studies, and aggregate-level data will be sought where IPD-level data are not available.
- As this is an observational study, it will not fully eliminate the possibility of confounding influences of unadjusted effects.
- However, unlike a randomised controlled trial, this study will provide a more comprehensive characterisation of temporal relationships between baseline androgen concentrations and health outcomes in community-dwelling adult males.

## Introduction

As men grow older, testosterone production and circulating concentrations of testosterone decline while comorbidities accumulate.^1–4^ Older men, even those in very good health, have lower circulating testosterone concentrations compared with healthy young men.^5 6^ Although results have been inconsistent, an increasing number of studies have reported associations of low endogenous testosterone concentration with poorer health outcomes, especially in older men. For instance, studies that have used liquid chromatography tandem mass spectrometry, widely regarded as the reference method for the measurement of total testosterone concentrations,^7^ have reported associations of lower endogenous testosterone concentrations with (1) cardiovascular disease and all-cause mortality in some cases^8–15^ but not others^8 16–20^; (2) some cancers but not others^21 22^ and (3) dementia^23 24^ but not laboratory measures of cognitive function.^25^ Therefore, it remains unclear whether testosterone is a biomarker of ill health or a causal factor for diseases of ageing.

Currently, testosterone treatment is recommended for men who have symptoms and signs of androgen deficiency and low testosterone concentrations, due to disease of the hypothalamus, pituitary or testes (organic or pathological hypogonadism).^26–29^ Randomised controlled trials of testosterone treatment in men aged 65 and older with low-normal testosterone concentrations without organic hypogonadism have shown modest benefits on sexual function, anaemia, self-reported physical function and mobility and volumetric bone density, but not on some objective measures of cognition over 12–36 months.^30–34^ The effect of testosterone on major adverse cardiovascular events (MACE) remains unclear.^35 36^ However, the selection criteria of these trials were such that the screening to enrolment ratio was 65:1, a highly selected population of older men.^32 33 36^ Importantly, the trials were neither large enough nor long enough to determine the effects of testosterone on MACE, development of dementia, bone fractures and mortality.^36^ Therefore, a meta-analysis of data from prospective cohort studies, with extended follow-up periods, provides opportunities for better understanding of the temporal profiles of the postulated associations between endogenous testosterone concentration and incident health outcomes. Meta-analyses of individual participant data (IPD) are generally preferable to meta-analyses of aggregate data (AD) in that they typically have higher statistical power and provide scope to control for important confounders and risk factors.^37 38^ Furthermore, one-stage IPD meta-analyses are preferable to two-stage approaches because the former uses an exact likelihood to directly model the distribution of IPD, offers the convenience of using a standard set of diagnostic tools to assess model fit and can arguably provide greater flexibility, in terms of options for statistical modelling.^39 40^

The Androgens In Men Study (AIMS), an international collaboration of prospective cohort studies, will examine the associations of sex hormones (comprising testosterone and dihydrotestosterone as the major androgens, and oestradiol as the major oestrogen, in the circulation) with health outcomes that are major sources of morbidity and mortality in middle-aged and older men. The group will perform a series of IPD meta-analyses to clarify the influence of sex hormone exposures on major health outcomes, including heart attack, stroke and cardiovascular deaths, cancer, new-onset dementia and all-cause mortality, as well as provide information on social, demographic and behavioural factors that are associated with endogenous testosterone concentrations. This work will characterise robustly the associations of several sex hormones with health outcomes in men in general over and above the study-specific estimates, and thus clarify the role of androgens as biomarkers for, or causal contributors to, men’s health. The work outlined in this document will be conducted from 1 February 2019 to 30 November 2020.

### Objectives

The AIMS will establish an international collaboration of existing cohort studies to clarify the relation of endogenous sex hormones with major health outcomes in men. ‘Population, exposure, outcomes’ characteristics include adult men in the general community; an exposure of endogenous circulating sex hormone concentration, primarily testosterone, as the principal male sex hormone or androgen; and a prospective cohort study type, with incident health outcomes including incidence of cardiovascular disease events, mortality, cancers and dementia. The specific objectives of the AIMS are to investigate associations between variables representing social, demographic and behavioural factors with the measured concentration of testosterone in the blood of men (Analysis 1); to examine the associations between testosterone concentrations and subsequent incidence of cardiovascular events, cardiovascular deaths and all-cause mortality in men (Analysis 2); to examine the associations between testosterone concentrations and subsequent mortality from (and, if available, diagnoses of) common cancers in men (Analysis 3) and to examine the associations between testosterone concentrations and cognitive impairment and incident dementia in men (Analysis 4). Analysis 2 will evaluate myocardial infarction, stroke and heart failure, deaths due to cardiovascular disease and the composite endpoint of MACE comprising non-fatal myocardial infarction, non-fatal stroke and deaths due to cardiovascular disease. Analysis 3 will evaluate outcomes of deaths due to and diagnoses of colorectal cancer, lung cancer and prostate cancer.

## Methods and analysis

IPD meta-analyses will be conducted to understand the associations between testosterone and a range of associated major health outcomes in men. IPD meta-analyses have been selected as the most suitable approach because (1) the required AD are not available from each of the cohorts in published literature; (2) IPD meta-analyses typically have higher statistical power than AD meta-analyses and provide scope for controlling for important confounders and risk factors.^37 38^ Where possible, methods will adhere to the guidelines for Preferred Reporting Items for Systematic Reviews and Meta-Analyses of IPD data (completed checklist is provided in online supplementary material) and the Meta-analysis Of Observational Studies in Epidemiology.^41–43^

10.1136/bmjopen-2019-034777.supp1 Supplementary data

### Studies selected for inclusion in IPD meta-analyses

Studies will be identified by two independent reviewers from a systematic review using online search tools for mainstream published (MEDLINE, EMBASE) and grey literature (OpenGrey, Mednar) studies, conducted from 14 June 2019 to 31 December 2019. Eligible studies include prospective cohort studies with plasma or serum testosterone concentrations measured using mass spectrometry with at least 5 years of follow-up data, with incident cardiovascular, cancer, mortality, dementia or cognitive events recorded (see online supplementary table S1 for an example of search criteria to be used). The search strategy selects for articles based on words or Medical Subject Headings terms matching the relevant exposure (example steps 1–3), outcomes (example steps 4–10) and study type (example steps 14–15), then, depending on the search tool, filters down to more relevant studies, with exclusions of clinical trials and of studies on non-humans (example steps 18–27), with no date range restrictions. Ahead of the systematic review, nine eligible studies (cohorts) had expressed interest to collaborate: three from Australia (Busselton Health Study,^44^ Health In Men Study,^45^ Men Androgen Inflammation Lifestyle Environment and Stress Study^46^); two from Europe (European Male Ageing Study,^47^ Osteoporotic Fractures in Men Study, Sweden^48^) and four from the USA (Atherosclerosis Risk in Communities Study,^49^ Cardiovascular Health Study,^50^ The Framingham Heart Study,^51^ Osteoporotic Fractures in Men Study, USA^52^). Investigators from eight of these studies have confirmed availability of suitable IPD-level data, provisional on approvals from their respective Publication and Steering Committees. If it is not possible to obtain IPD-level data from selected studies, we will request suitable AD-level data.

10.1136/bmjopen-2019-034777.supp2 Supplementary data

### Data provision, merging, harmonisation and storage

The project manager will liaise directly with the nominated contact person for each cohort study to identify, specifically, which variables and set of observations will be suitable to request. A data request will then be submitted to the data custodian for each study. Requested variables will be labelled as either ‘highly desirable’ or ‘only if available’, in order to prioritise efforts in obtaining the key variables for analyses and to acknowledge the differing availability of variables among studies. A list of variable names, definitions and attributes, including numbers of rows and columns in each data file(s) will also be requested to be provided separately. Methods for ascertaining outcome and comorbidity status will be requested, which can be used to indicate the relative quality of diagnostic information (eg, International Classification of Diseases (ICD)-coded diagnoses from hospital inpatient admissions vs self-report information). File transfer will be achieved via encrypted file transfer (or other sufficiently secure method).

Once each dataset has been received by the project manager, the original file(s) will be saved and date-stamped in a secure central repository. All subsequent manipulations will be completed using copies of the original file, with syntax saved as script files. Variable definitions will be checked, variables inspected for missing values and variable properties and value ranges assessed to identify possible outliers. A table of summary statistics will be calculated and, where possible, analyses run and compared with published values to check for data consistency. The nature of any discrepancies identified from these checks will need to be understood, and possibly resolved, prior to proceeding with the meta-analyses.^42 53 54^

Depending on the extent of missing-ness, missing data will be suitably imputed.^54 55^ For each analysis, we will conduct multiple imputations using a method that approximates as close as possible to the substantive model.^56^ The method will likely vary depending on the analysis^57^ and therefore we will consider adapting either a fully conditional specification^58^ or joint modelling framework^59^ to each case, as is appropriate. The quality of imputations will be quantified by using re-imputed values to calculate the posterior predictive p values of relevant quantities.^60^ Results from each of the multiply-imputed datasets will be suitably pooled to obtain final estimates, SEs and 95% CIs.^61^

Prior to the merging of datasets, a variable to identify each source study will be appended as the new first column. Variable formats will be checked and corrected for consistency and participant identifier codes anonymised for uniqueness across all studies. Harmonisation will be required for some variables (eg, physical activity, alcohol consumption) that are recorded differently by the different component studies. Where possible, AD datasets will be used to reconstruct IPD-level data (that is, partially reconstructed IPD) prior to merging.^62–64^ Should this not be possible, the IPD-level data will be aggregated and summary estimates made with and without AD-level data as sensitivity analyses. It is also possible that some studies might have outcomes available for some analyses, but not others; in these cases, we will preferentially use IPD-level data when available but also seek to use AD-level data from those other studies when available. There is no requirement to use IPD-level data from the same studies across all analyses.

All IPD-level data will be accessible to only the approved staff from a secure on-site facility, from rooms that are kept locked when unattended and with remote access not permitted. IPD-level data are not to be printed in hard copy and will only be presented at aggregate level. Data analysed for this study will be retained for 5 years after the last of all proposed analyses are published and will then be destroyed. A post-analysis retention period is required to enable publication and possible scrutiny of findings. Disposal will be carried out according to best practice.

### Data items

The full list of generic variables to include in meta-analyses is presented in table 1. Analysis 1 will model relationships of total testosterone concentrations, as measured in serum or plasma samples (dependent variable), with key demographic variables and risk factors for disease (predictor variables). Time-to-event variables obtained from follow-up data will be analysed in Analysis 2 and 3 (outcomes), and their associations with testosterone concentrations (focal predictor) and other potential confounders and risk factors (predictor variables). Records of dementia diagnoses (physician or otherwise categorised) and relative cognitive function (for example, from test scores) will be analysed in Analysis 4, and their associations with testosterone concentrations and other potential confounders and risk factors. Analyses of relationships with other sex hormones instead of testosterone, including dihydrotestosterone, oestradiol, luteinising hormone (LH) and sex hormone binding globulin (SHBG), will be conducted where sufficient data are available. For exploratory analyses, free testosterone, the amount in the circulation which is not protein-bound, will be calculated from measured total testosterone and SHBG.^65^ Covariates were selected to include those used in previous studies, as well as those that are typically recorded. The full list of proposed variables to include in the meta-analyses is presented in table 1.

**Table 1:** Variables planned to be included in IPD meta-analysis modelling*

Type	Analysis 1	Analysis 2	Analysis 3	Analysis 4
Outcomes/DV	Androgen concentration†	Incident CVD‡	Incident deaths (cancer‡)	Incident dementia
		Incident deaths (CVD‡)	Incident cancer‡ (diagnoses)	Baseline cognition
		Incident deaths (all-cause)		Change in cognition
Focal predictor	–	Androgen concentration†	Androgen concentration†	Androgen concentration†
Covariates/IV demographic	Age	Age	Age	Age
	Education level	Education level	Education level	Education level
	Ethnicity	Ethnicity	Ethnicity	Ethnicity
	Marital status	Marital status	Marital status	Marital status
	Site	Site	Site	Site
Risk factors and comorbidities	Alcohol consumption	Alcohol consumption	Alcohol consumption	Alcohol consumption
	BMI, waist	BMI, waist	BMI, waist	BMI, waist
	Physical activity	Physical activity	Physical activity	Physical activity
	Smoking status	Smoking status	Smoking status	Smoking status
	BP, hypertension	BP, hypertension	BP, hypertension	BP, hypertension
	General health	General health	General health	General health
		Atrial fibrillation		
	Prevalent CVD	Prevalent CVD		
	Prevalent cancer‡		Prevalent cancer‡	
	Prevalent dementia			Prevalent dementia
	Baseline cognition			
	COPD	COPD		
	Diabetes	Diabetes	Diabetes	Diabetes
	Cholesterol, LDL, HDL	Cholesterol, LDL, HDL		
	Creatinine level	Creatinine level		
	Lipid lowering medications	Lipid lowering medications		
	Anxiety			Anxiety
	Depression			Depression
	Psychotropic drug use			Psychotropic drug use

*Black font: highly desirable; Green font: only if available.

†Androgens: total testosterone, dihydrotestosterone, oestradiol, luteinising hormone, sex hormone binding globulin.

‡Subgroup analyses are also planned. For CVD outcomes: heart failure, myocardial infarction, stroke. For cancer outcomes: colorectal cancer, lung cancer, prostate cancer.

BMI, body mass index; BP, blood pressure; COPD, chronic obstructive pulmonary disease; CVD, cardiovascular disease; DV, dependent variable; IV, independent variables.

### Statistical analysis

Since the datasets to be analysed are sampled from different populations, a random-effects meta-analysis is appropriate, as it acknowledges that effects will vary among studies due to differences in local factors.^66^ One-stage IPD meta-analyses will be performed. Each IPD meta-analysis will involve fitting a model with study estimated as either a fixed or random term, to account for related observations within studies, and testosterone modelled as random slopes (when modelled as a continuous predictor) or intercepts (when modelled as a categorical predictor), to harmonised, merged data from all cohorts. The underlying statistical model and estimates of effect size will be specific to each of the proposed analyses and are outlined as follows.

#### Analysis 1: factors associated with testosterone concentrations in men and characterisation of reference ranges

Linear mixed models (LMMs) or generalised linear mixed models (GLMMs) will be used to model the relation between the predictors (independent variables) and each hormonal variable (testosterone, dihydrotestosterone, oestradiol, LH and SHBG) as five separate IPD meta-analyses. Suspected non-linear relationships, at the scale of the linear predictor, will be investigated and modelled appropriately (eg, splines with pre-set knot locations and linear boundary constraints). Measures of effect size may include (but are not limited to): η^2^,^67 68^ Pearson’s r, standardised mean difference to the reference level (categorical predictors) and standardised difference for an increase in one SD (continuous predictors). In the case of non-linear relations, we will graphically describe the relationship with comparisons made with appropriate reference points. Reference ranges will be derived based on the distributions of testosterone in healthy men.

#### Analysis 2: associations between testosterone concentrations and subsequent incidence of cardiovascular events, cardiovascular deaths and all-cause mortality

Cox proportional hazards models will be used to assess the effect of testosterone level on the incident risk of each outcome, with separate IPD meta-analyses conducted for each outcome (myocardial infarction, stroke, heart failure, deaths due to cardiovascular disease and MACE) and each hormonal variable (testosterone, dihydrotestosterone, oestradiol, LH and SHBG). Component study will be modelled either as a stratified variable^39^ or as a random term, and testosterone as a random term, using frailty models, which are a class of survival models that incorporate random effects.^69–71^ Participants with prevalent cardiovascular disease at baseline will be excluded. The length of follow-up will also be standardised among studies in order to maximise data from all datasets, while minimising the prospect for variable lengths-to-follow-up among studies introducing additional heterogeneity into results.^55^

Multivariable versions of each of these models will also be fitted, with additional predictors for potential confounders and risk factors included (table 1). Non-linear associations for continuous variables will be modelled using natural splines with pre-specified knots and linear boundary constraints. The (standardised) measure of effect size used will be the HR. Subgroup analyses will be conducted separately for each of three specific types of cardiovascular disease (outcomes): myocardial infarction, stroke and heart failure.

#### Analysis 3: association between testosterone level and subsequent incidence of cancer

Cox proportional hazards models will be used to assess the effect of testosterone concentrations on the incident risk of cancer deaths and, if available, of cancer diagnoses. IPD meta-analyses will be conducted separately for each of these outcomes and for each hormonal variable as focal predictor (testosterone, dihydrotestoterone, oestradiol, LH and SHBG). Component study will be modelled either as a stratified variable^39^ or as a random term, and testosterone as a random term, using frailty models.^69–71^ Participants with prevalent cancer diagnosis at baseline will be excluded. The length of follow-up will be standardised among studies in order to maximise data from all datasets, while minimising the prospect for variable lengths-to-follow-up among studies introducing additional heterogeneity into results.^55^

Multivariable versions of each of these models will also be fitted, with additional predictors for potential confounders and risk factors included (table 1). Non-linear associations for continuous variables will be modelled using natural splines with pre-specified knots and linear boundary constraints. The (standardised) measure of effect size used will be the HR. Subgroup analyses will be conducted separately for each of three common types of cancers in men (outcomes): colorectal cancer, lung cancer and prostate cancer. For these analyses, men with the relevant cancer type at baseline will be excluded from that specific analysis. Thus men with prevalent colorectal cancer (but not other cancer types) will be excluded in the analysis of incident colorectal cancer; similarly for the analyses of incident lung and prostate cancer, men with lung or prostate cancer at baseline will be excluded.

#### Analysis 4: associations of testosterone levels with cognitive impairment and incident dementia in men

LMMs and GLMMs will be used to model the association of testosterone concentrations with cognitive impairment (cross-sectional analyses of baseline data). Cox proportional hazards models will be used to assess the effect of testosterone concentrations on the incident risk of dementia. Men with prevalent dementia will be excluded from this analysis. We will also ask for follow-up cognition test scores, and if available, will run an analysis of changes in cognition test scores from baseline as an outcome. Separate IPD meta-analyses will be conducted for each hormonal variable as a focal predictor (testosterone, dihydrotestosterone, oestradiol, LH and SHBG). Component study will be modelled as a fixed or random intercept term in LMMs and GLMMs and as a stratified factor or frailty model random term in Cox regressions. Multivariable versions of each of these models will also be fitted, with additional predictors for potential confounders and risk factors included (table 1). Suspected non-linear relationships, at the scale of the linear predictor, will be investigated and modelled using splines with pre-specified knots and linear boundary constraints. Measures of effect size may include: η^2^, Pearson’s r, standardised mean difference to the reference level (categorical predictors), standardised difference for an increase in one SD (continuous predictors), OR for LMMs and GLMMs and HR for Cox regressions.

Throughout all analyses, contour-enhanced funnel plots will be constructed to visually assess patterns in estimates of effect sizes and precision among studies, to investigate heterogeneity and possible meta-biases.^72–74^ The relative amount of heterogeneity will be estimated (eg, using I^2^) and forest plots presented. Subgroup or meta-regression analyses may be conducted if pronounced heterogeneity is estimated.^66^

### Patient and public involvement

This IPD meta-analysis will use existing secondary data. Patients and public were not involved in the design, recruitment or conduct of this IPD meta-analysis. The results of this study will be shared with the primary investigators of the shared studies and disseminated as publications in open-access journals.

## Ethics and dissemination

Ethics approvals obtained for each of the participating cohorts state that participants have consented to have their data collected and used for research purposes. Furthermore, there are no expected harms or risks to participants, as data have already been collected within each individual cohort, under existing ethics approvals. De-identified data will be collated and analysed, with no new procedures planned for participants. The AIMS has been assessed as exempt from ethics review by the Human Research Ethics Office at the University of Western Australia (file reference number RA/4/20/5014). Research findings will be disseminated to the scientific and broader community via the publication of the four planned research articles, at scientific conferences and meetings of relevant professional societies (including the Endocrine Society) to ensure the clinical translation and uptake of findings. Collaborating cohort studies each have their own individual policies and strategies in place for the dissemination of findings to study participants and local communities.

## Discussion

Although several published meta-analyses have investigated associations of endogenous testosterone with health outcomes in men,^75–86^ none have conducted IPD meta-analyses of health outcomes as planned for this study. A previous IPD meta-analysis focussed on the outcome of metabolic syndrome.^78^ Results from this study will improve on previously published estimates from individual studies, in terms of the generalisability of findings. Estimates from the IPD meta-analyses are also likely to be more reliable than those published from conventional meta-analyses because they typically have higher statistical power and provide scope for controlling for important confounders and risk factors.^37 38^ Uncertainty will undoubtedly remain due to the possibility of confounding influences of unadjusted effects. However, unlike a randomised controlled trial, this study avoids the need to subject individuals to interventions, and provides more comprehensive characterisation of temporal relationships between baseline testosterone concentrations and a range of key health outcomes.^87^

Accordingly, it is hoped that the AIMS collaboration will ultimately complement the research efforts and outputs from multiple prospective cohort studies by drawing on the collective body of evidence to clarify the role of endogenous sex hormone levels on major health outcomes in men. It is possible that this work might also elucidate new understanding, arising from improved scope for fitting more complex models due to increased statistical power or from patterns detected in subgroup or meta-regression analyses. Clinically, research outputs will be used to identify the scope and optimal recruitment criteria for future trials of testosterone therapy. These data will also allow reference ranges for testosterone in men across ages and geographical locations to be refined, to inform recommendations for clinical practice more generally.

## Supplementary Material

Reviewer comments

Author's manuscript

## References

[1] Handelsman DJ, Yeap BB, Flicker L, et al Age-Specific population centiles for androgen status in men. Eur J Endocrinol 2015;173:809–17. 10.1530/EJE-15-0380 26385186

[2] Hsu B, Cumming RG, Hirani V, et al Temporal trend in androgen status and androgen-sensitive outcomes in older men. J Clin Endocrinol Metab 2016;101:1836–46. 10.1210/jc.2015-3810 26918290

[3] Shi Z, Araujo AB, Martin S, et al Longitudinal changes in testosterone over five years in community-dwelling men. J Clin Endocrinol Metab 2013;98:3289–97. 10.1210/jc.2012-3842 23775354

[4] Yeap BB, Manning L, Chubb SAP, et al Progressive impairment of testicular endocrine function in ageing men: testosterone and dihydrotestosterone decrease, and luteinizing hormone increases, in men transitioning from the 8th to 9th decades of life. Clin Endocrinol 2018;88:88–95. 10.1111/cen.13484 28945276

[5] Sikaris K, McLachlan RI, Kazlauskas R, et al Reproductive hormone reference intervals for healthy fertile young men: evaluation of automated platform assays. J Clin Endocrinol Metab 2005;90:5928–36. 10.1210/jc.2005-0962 16118337

[6] Yeap BB, Alfonso H, Chubb SAP, et al Reference ranges and determinants of testosterone, dihydrotestosterone, and estradiol levels measured using liquid chromatography-tandem mass spectrometry in a population-based cohort of older men. J Clin Endocrinol Metab 2012;97:4030–9. 10.1210/jc.2012-2265 22977273

[7] Handelsman DJ, Wartofsky L Requirement for mass spectrometry sex steroid assays in the Journal of clinical endocrinology and metabolism. J Clin Endocrinol Metab 2013;98:3971–3. 10.1210/jc.2013-3375 24098015

[8] Hsu B, Cumming RG, Naganathan V, et al Temporal changes in androgens and estrogens are associated with all-cause and cause-specific mortality in older men. J Clin Endocrinol Metab 2016;101:2201–10. 10.1210/jc.2016-1025 26963953

[9] Ohlsson C, Barrett-Connor E, Bhasin S, et al High serum testosterone is associated with reduced risk of cardiovascular events in elderly men. J Am Coll Cardiol 2011;58:1674–81. 10.1016/j.jacc.2011.07.019 21982312

[10] Pye SR, Huhtaniemi IT, Finn JD, et al Late-Onset hypogonadism and mortality in aging men. J Clin Endocrinol Metab 2014;99:1357–66. 10.1210/jc.2013-2052 24423283

[11] Tivesten Åsa, Mellström D, Jutberger H, et al Low serum testosterone and high serum estradiol associate with lower extremity peripheral arterial disease in elderly men. J Am Coll Cardiol 2007;50:1070–6. 10.1016/j.jacc.2007.04.088 17825717

[12] Tivesten Åsa, Vandenput L, Labrie F, et al Low serum testosterone and estradiol predict mortality in elderly men. J Clin Endocrinol Metab 2009;94:2482–8. 10.1210/jc.2008-2650 19401373

[13] Yeap BB, Alfonso H, Chubb SAP, et al In older men, higher plasma testosterone or dihydrotestosterone is an independent predictor for reduced incidence of stroke but not myocardial infarction. J Clin Endocrinol Metab 2014;99:4565–73. 10.1210/jc.2014-2664 25268392

[14] Yeap BB, Hyde Zoë, Almeida OP, et al Lower testosterone levels predict incident stroke and transient ischemic attack in older men. J Clin Endocrinol Metab 2009;94:2353–9. 10.1210/jc.2008-2416 19351733

[15] Yeap BB, Knuiman MW, Divitini ML, et al Differential associations of testosterone, dihydrotestosterone and oestradiol with physical, metabolic and health-related factors in community-dwelling men aged 17-97 years from the Busselton Health Survey. Clin Endocrinol 2014;81:100–8. 10.1111/cen.12407 24428256

[16] Chan YX, Knuiman MW, Hung J, et al Neutral associations of testosterone, dihydrotestosterone and estradiol with fatal and non-fatal cardiovascular events, and mortality in men aged 17-97 years. Clin Endocrinol 2016;85:575–82. 10.1111/cen.13089 27106765

[17] Shores MM, Arnold AM, Biggs ML, et al Testosterone and dihydrotestosterone and incident ischaemic stroke in men in the cardiovascular health study. Clin Endocrinol 2014;81:746–53. 10.1111/cen.12452 PMC4169352 24645738

[18] Shores MM, Biggs ML, Arnold AM, et al Testosterone, dihydrotestosterone, and incident cardiovascular disease and mortality in the cardiovascular health study. J Clin Endocrinol Metab 2014;99:2061–8. 10.1210/jc.2013-3576 24628549 PMC4037728

[19] Srinath R, Hill Golden S, Carson KA, et al Endogenous testosterone and its relationship to preclinical and clinical measures of cardiovascular disease in the Atherosclerosis risk in Communities study. J Clin Endocrinol Metab 2015;100:1602–8. 10.1210/jc.2014-3934 25584720 PMC5393511

[20] Srinath R, Gottesman RF, Hill Golden S, Golden SH, et al Association between endogenous testosterone and cerebrovascular disease in the ARIC study (atherosclerosis risk in communities). Stroke 2016;47:2682–8. 10.1161/STROKEAHA.116.014088 27729576

[21] Chan YX, Knuiman MW, Divitini ML, et al Lower Circulating Androgens Are Associated with Overall Cancer Risk and Prostate Cancer Risk in Men Aged 25–84 Years from the Busselton Health Study. Horm Canc 2018;9:391–8. 10.1007/s12672-018-0346-5 PMC10355941 30097782

[22] Daniels NA, Nielson CM, Hoffman AR, et al Sex hormones and the risk of incident prostate cancer. Urology 2010;76:1034–40. 10.1016/j.urology.2010.01.086 20451981 PMC2972405

[23] Ford AH, Yeap BB, Flicker L, et al Sex hormones and incident dementia in older men: the health in men study. Psychoneuroendocrinology 2018;98:139–47. 10.1016/j.psyneuen.2018.08.013 30144781

[24] Ford AH, Yeap BB, Flicker L, et al Prospective longitudinal study of testosterone and incident depression in older men: the health in men study. Psychoneuroendocrinology 2016;64:57–65. 10.1016/j.psyneuen.2015.11.012 26615472

[25] LeBlanc ES, Wang PY, Janowsky JS, et al Association between sex steroids and cognition in elderly men. Clin Endocrinol 2010;72:393–403. 10.1111/j.1365-2265.2009.03692.x PMC2852485 19744108

[26] Yeap BB, Wu FCW Clinical practice update on testosterone therapy for male hypogonadism: contrasting perspectives to optimize care. Clin Endocrinol 2019;90:56–65. 10.1111/cen.13888 30358898

[27] Yeap BB, Grossmann M, McLachlan RI, et al Endocrine Society of Australia position statement on male hypogonadism (Part 2): treatment and therapeutic considerations. Med J Aust 2016;205:228–31. 10.5694/mja16.00448 27581270

[28] Yeap BB, Grossmann M, McLachlan RI, et al Endocrine Society of Australia position statement on male hypogonadism (Part 1): assessment and indications for testosterone therapy. Med J Aust 2016;205:173–8. 10.5694/mja16.00393 27510348

[29] Bhasin S, Brito JP, Cunningham GR, et al Testosterone therapy in men with hypogonadism: an endocrine Society* clinical practice guideline. J Clin Endocrinol Metab 2018;103:1715–44. 10.1210/jc.2018-00229 29562364

[30] Resnick SM, Matsumoto AM, Stephens-Shields AJ, et al Testosterone treatment and cognitive function in older men with low testosterone and age-associated memory impairment. JAMA 2017;317:717–27. 10.1001/jama.2016.21044 28241356 PMC5433758

[31] Roy CN, Snyder PJ, Stephens-Shields AJ, et al Association of testosterone levels with anemia in older men: a controlled clinical trial. JAMA Intern Med 2017;177:480–90. 10.1001/jamainternmed.2016.9540 28241237 PMC5433757

[32] Snyder PJ, Bhasin S, Cunningham GR, et al Effects of testosterone treatment in older men. N Engl J Med 2016;374:611–24. 10.1056/NEJMoa1506119 26886521 PMC5209754

[33] Snyder PJ, Bhasin S, Cunningham GR, et al Lessons from the testosterone trials. Endocr Rev 2018;39:369–86. 10.1210/er.2017-00234 29522088 PMC6287281

[34] Snyder PJ, Kopperdahl DL, Stephens-Shields AJ, et al Effect of testosterone treatment on volumetric bone density and strength in older men with low testosterone. JAMA Intern Med 2017;177:471–9. 10.1001/jamainternmed.2016.9539 28241231 PMC5433755

[35] Budoff MJ, Ellenberg SS, Lewis CE, et al Testosterone treatment and coronary artery plaque volume in older men with low testosterone. JAMA 2017;317:708–16. 10.1001/jama.2016.21043 28241355 PMC5465430

[36] Yeap BB, Page ST, Grossmann M Testosterone treatment in older men: clinical implications and unresolved questions from the testosterone trials. Lancet Diabetes Endocrinol 2018;6:659–72. 10.1016/S2213-8587(17)30416-3 30017800

[37] Riley RD, Lambert PC, Abo-Zaid G Meta-Analysis of individual participant data: rationale, conduct, and reporting. BMJ 2010;340:c221 10.1136/bmj.c221 20139215

[38] Stewart LA, Parmar MKB Meta-Analysis of the literature or of individual patient data: is there a difference? Lancet 1993;341:418–22. 10.1016/0140-6736(93)93004-K 8094183

[39] Burke DL, Ensor J, Riley RD Meta-Analysis using individual participant data: one-stage and two-stage approaches, and why they may differ. Stat Med 2017;36:855–75. 10.1002/sim.7141 27747915 PMC5297998

[40] Debray TPA, Moons KGM, Abo-Zaid GMA, et al Individual participant data meta-analysis for a binary outcome: one-stage or two-stage? PLoS One 2013;8:e60650 10.1371/journal.pone.0060650 23585842 PMC3621872

[41] Munn Z, Stern C, Aromataris E, et al What kind of systematic review should I conduct? A proposed typology and guidance for systematic reviewers in the medical and health sciences. BMC Med Res Methodol 2018;18 10.1186/s12874-017-0468-4 PMC5761190 29316881

[42] Stewart LA, Clarke M, Rovers M, et al Preferred reporting items for a systematic review and meta-analysis of individual participant data. JAMA 2015;313:1657–65. 10.1001/jama.2015.3656 25919529

[43] Stroup DF, Berlin JA, Morton SC, et al Meta-Analysis of observational studies in epidemiology. JAMA 2000;283:2008–12. 10789670 10.1001/jama.283.15.2008

[44] Knuiman MW, Jamrozik K, Welborn TA, et al Age and secular trends in risk factors for cardiovascular disease in Busselton. Aust J Public Health 1995;19:375–82. 10.1111/j.1753-6405.1995.tb00389.x 7578538

[45] Norman PE, Flicker L, Almeida OP, et al Cohort profile: the health in men study (HIMS). Int J Epidemiol 2009;38:48–52. 10.1093/ije/dyn041 18316347

[46] Grant JF, Martin SA, Taylor AW, et al Cohort profile: the men androgen inflammation lifestyle environment and stress (MAILES) study. Int J Epidemiol 2014;43:1040–53. 10.1093/ije/dyt064 23785097 PMC4258764

[47] Lee DM, O’Neill TW, Pye SR, et al The European male ageing study (EMAS): design, methods and recruitment. Int J Androl 2009;32:11–24. 10.1111/j.1365-2605.2008.00879.x 18328041

[48] Mellström D, Johnell O, Ljunggren Östen, et al Free testosterone is an independent predictor of BMD and prevalent fractures in elderly men: MROS Sweden. J Bone Miner Res 2006;21:529–35. 10.1359/jbmr.060110 16598372

[49] The ARIC Investigators The Atherosclerosis risk in communities (ARIC) study: design and objectives. Am J Epidemiol 1989;129:687–702. 10.1093/oxfordjournals.aje.a115184 2646917

[50] Fried LP, Borhani NO, Enright P, et al The cardiovascular health study: design and rationale. Ann Epidemiol 1991;1:263–76. 10.1016/1047-2797(91)90005-W 1669507

[51] Kannel WB, Feinleib M, McNamara PM, et al An investigation of coronary heart disease in families. The Framingham offspring study. Am J Epidemiol 1979;110:281–90. 10.1093/oxfordjournals.aje.a112813 474565

[52] Orwoll E, Blank JB, Barrett-Connor E, et al Design and baseline characteristics of the osteoporotic fractures in men (MROS) study — a large observational study of the determinants of fracture in older men. Contemp Clin Trials 2005;26:569–85. 10.1016/j.cct.2005.05.006 16084776

[53] Higgins JPT, Altman DG, Sterne JAC Chapter 8: Assessing risk of bias in included studies : Higgins JPT, Altman DG, Sterne JAC, Cochrane Handbook for systematic reviews of interventions version 520. The Cochrane Collaboration, 2017.

[54] Liberati A, Altman DG, Tetzlaff J, et al The PRISMA statement for reporting systematic reviews and meta-analyses of studies that evaluate health care interventions: explanation and elaboration. PLoS Med 2009;6:e1000100 10.1371/journal.pmed.1000100 19621070 PMC2707010

[55] Deeks JJ, Higgins JPT, Altman DG Chapter 9: Analysing data and undertaking meta-analyses : Higgins JPT, Churchill R, Chandler J, et al, Cochrane Handbook for systematic reviews of interventions version 520. The Cochrane Collaboration, 2017.

[56] van Buuren S Flexible imputation of missing data. 2nd edn Milton: CRC Press LLC, 2018.

[57] Graham JW Missing data analysis: making it work in the real world. Annu Rev Psychol 2009;60:549–76. 10.1146/annurev.psych.58.110405.085530 18652544

[58] Van Buuren S, Brand JPL, Groothuis-Oudshoorn CGM, et al Fully conditional specification in multivariate imputation. J Stat Comput Simul 2006;76:1049–64. 10.1080/10629360600810434

[59] Kenward M, Carpenter J Multiple imputation of quantitative data. multiple imputation and its application. Chicester: Wiley, 2013: 77–89.

[60] He Y, Zaslavsky AM Diagnosing imputation models by applying target analyses to posterior replicates of completed data. Stat Med 2012;31:1–18. 10.1002/sim.4413 22139814 PMC4233994

[61] Rubin DB Multiple imputation for nonresponse in surveys. New York: Wiley, 1987.

[62] Hukkelhoven CWPM, Steyerberg EW, Rampen AJJ, et al Patient age and outcome following severe traumatic brain injury: an analysis of 5600 patients. J Neurosurg 2003;99:666–73. 10.3171/jns.2003.99.4.0666 14567601

[63] Riley RD, Simmonds MC, Look MP Evidence synthesis combining individual patient data and aggregate data: a systematic review identified current practice and possible methods. J Clin Epidemiol 2007;60:431.e1–9. 10.1016/j.jclinepi.2006.09.009 17419953

[64] Steyerberg EW, Kievit J, de Mol Van Otterloo JC, et al Perioperative mortality of elective abdominal aortic aneurysm surgery. A clinical prediction rule based on literature and individual patient data. Arch Intern Med 1995;155:1998–2004. 7575054

[65] Ly LP, Sartorius G, Hull L, et al Accuracy of calculated free testosterone formulae in men. Clin Endocrinol 2010;73:382–8. 10.1111/j.1365-2265.2010.03804.x 20346001

[66] Borenstein M, Hedges LV, Higgins JPT, et al Introduction to meta-analysis. West Sussex: John Wiley & Sons Ltd, 2009.

[67] Fisher R Statistical methods for research workers. 2nd edn New York: Hafner, 1928.

[68] Pearson K On a correction needful in the case of the correlation ratio. Biometrika 1911;8:254–6.

[69] Keiding N, ANDERSEN PERK, Klein JP The role of frailty models and accelerated failure time models in describing heterogeneity due to omitted covariates. Stat Med 1997;16:215–24. 10.1002/(SICI)1097-0258(19970130)16:2&lt;215::AID-SIM481&gt;3.0.CO;2-J 9004393

[70] Vaupel JW, Manton KG, Stallard E The impact of heterogeneity in individual frailty on the dynamics of mortality. Demography 1979;16:439–54. 10.2307/2061224 510638

[71] Yashin AI, Vaupel JW, Iachine IA Correlated individual frailty: an advantageous approach to survival analysis of bivariate data. Math Popul Stud 1995;5:145–59. 10.1080/08898489509525394 12290053

[72] Ahmed I, Sutton AJ, Riley RD Assessment of publication bias, selection bias, and unavailable data in meta-analyses using individual participant data: a database survey. BMJ 2011;344:d7762 10.1136/bmj.d7762 22214758

[73] Shamseer L, Moher D, Clarke M, et al Preferred reporting items for systematic review and meta-analysis protocols (PRISMA-P) 2015: elaboration and explanation. BMJ 2015;349:g7647 10.1136/bmj.g7647 25555855

[74] Sterne JAC, Sutton AJ, Ioannidis JPA, et al Recommendations for examining and interpreting funnel plot asymmetry in meta-analyses of randomised controlled trials. BMJ 2011;343:d4002. 10.1136/bmj.d4002 21784880

[75] Araujo AB, Dixon JM, Suarez EA, et al Clinical review: endogenous testosterone and mortality in men: a systematic review and meta-analysis. J Clin Endocrinol Metab 2011;96:3007–19. 10.1210/jc.2011-1137 21816776 PMC3200249

[76] Atlantis E, Fahey P, Cochrane B, et al Endogenous testosterone level and testosterone supplementation therapy in chronic obstructive pulmonary disease (COPD): a systematic review and meta-analysis. BMJ Open 2013;3:e003127 10.1136/bmjopen-2013-003127 PMC3740247 23943774

[77] Boyle P, Koechlin A, Bota M, et al Endogenous and exogenous testosterone and the risk of prostate cancer and increased prostate-specific antigen (PSA) level: a meta-analysis. BJU Int 2016;118:731–41. 10.1111/bju.13417 26779889

[78] Brand JS, Rovers MM, Yeap BB, et al Testosterone, sex hormone-binding globulin and the metabolic syndrome in men: an individual participant data meta-analysis of observational studies. PLoS One 2014;9:e100409 10.1371/journal.pone.0100409 25019163 PMC4096400

[79] Brand JS, van der Tweel I, Grobbee DE, et al Testosterone, sex hormone-binding globulin and the metabolic syndrome: a systematic review and meta-analysis of observational studies. Int J Epidemiol 2011;40:189–207. 10.1093/ije/dyq158 20870782

[80] Claps M, Petrelli F, Caffo O, et al Testosterone levels and prostate cancer prognosis: systematic review and meta-analysis. Clin Genitourin Cancer 2018;16:165–75. 10.1016/j.clgc.2018.01.005 29454638

[81] Corona G, Monami M, Rastrelli G, et al Testosterone and metabolic syndrome: a Meta‐Analysis study. J Sex Med 2011;8:272–83. 10.1111/j.1743-6109.2010.01991.x 20807333

[82] Haddad RM, Kennedy CC, Caples SM, et al Testosterone and cardiovascular risk in men: a systematic review and meta-analysis of randomized placebo-controlled trials. Mayo Clinic Proc 2007;82:29–39. 10.1016/S0025-6196(11)60964-6 17285783

[83] Holmegard HN, Nordestgaard BG, Jensen GB, et al Sex hormones and ischemic stroke: a prospective cohort study and meta-analyses. J Clin Endocrinol Metab 2016;101:69–78. 10.1210/jc.2015-2687 26509870

[84] Isidori AM, Giannetta E, Gianfrilli D, et al Effects of testosterone on sexual function in men: results of a meta-analysis. Clin Endocrinol 2005;63:381–94. 10.1111/j.1365-2265.2005.02350.x 16181230

[85] Lv W, Du N, Liu Y, et al Low Testosterone Level and Risk of Alzheimer’s Disease in the Elderly Men: a Systematic Review and Meta-Analysis. Mol Neurobiol 2016;53:2679–84. 10.1007/s12035-015-9315-y 26154489

[86] Ruige JB, Mahmoud AM, De Bacquer D, et al Endogenous testosterone and cardiovascular disease in healthy men: a meta-analysis. Heart 2011;97:870–5. 10.1136/hrt.2010.210757 21177660

[87] Gordis L Epidemiology. 4th edn Philadelphia: Saunders, 2009.

