# Supplementary material for: Androgens In Men Study (AIMS): protocol for meta-analyses of individual participant data investigating associations of androgens with health outcomes in men
Source: BMJ Open. 2020 May 11;10(5):e034777. doi: 10.1136/bmjopen-2019-034777 (PMC7239545; doi:10.1136/bmjopen-2019-034777)
Supplement: Supplementary data [file bmjopen-2019-034777supp002.pdf]

Supplementary Table S1. Example search strategy for systematic review: AIMS study.

The following is the search strategy for MEDLINE. The search strategy for EMBASE, OpenGrey, and Mednar attempts to replicate this as closely as possible, and will be subsequently reported upon completion of the Systematic Review.

1. Testosterone/ or Androgens/
2. (testosterone or androgen* or sex hormone* or sex steroid*).ti.
3. (testosterone or androgen*).ab.
4. cardiovascular diseases/ or heart diseases/ or heart failure/ or vascular diseases/ or stroke/ or myocardial infarction/ or coronary disease/ or cerebrovascular disorders/
5. (cardiovascular or stroke or myocardial infarction or heart failure).ti.
6. neoplasms/ or colorectal neoplasms/ or lung neoplasms/ or prostatic neoplasms/
7. cancer.ti.
8. mortality/ or mortality.ti.
9. dementia/ or cognition/ or dementia.ti. or cognit*.ti.
10. Aging/psychology or Neuropsychological Tests/
11. 1 or 2 or 3
12. 4 or 5 or 6 or 7 or 8 or 9 or 10
13. 11 and 12
14. longitudinal studies/ or prospective studies/ or follow-up studies/ or cohort studies/
15. (prospective or follow-up or cohort study or longitudinal study).ti,ab.
16. 14 or 15
17. 13 and 16
18. (exogenous or replacement or therapy or hormone treatment).ti.
19. Hormone Replacement Therapy/
20. 18 or 19
21. 17 not 20
22. limit 21 to humans
23. limit 22 to english language
24. limit 23 to (adaptive clinical trial or address or autobiography or bibliography or biography or case reports or clinical trial, all or clinical trial, phase i or clinical trial, phase ii or clinical trial, phase iii or clinical trial, phase iv or clinical trial, veterinary or clinical trials, veterinary as topic or clinical trial protocol or clinical trial or comment or controlled clinical trial or dictionary or editorial or lecture or legislation or meta analysis or practice guideline or pragmatic clinical trial or published erratum or randomized controlled trial or retracted publication or "retraction of publication" or "review" or "scientific integrity review" or "systematic review")
25. Retrospective Studies/ or Case-Control Studies/ or (retrospective analysis or case-control).ti.
26. 24 or 25
27. 23 not 26

<u>Notes:</u>

Terms with a trailing “/” are MeSH terms and those with a trailing “*” are truncated search strings. This search strategy is included with the PROSPERO registration for the AIMS

project (Registration No. CRD42019139668). Beforehand, a search of PROSPERO was conducted for another suitable strategy but none were found. However, the above strategy is based upon one that has been used for a similar study.[1]

References cited

1. Holmegard HN, Nordestgaard BG, Jensen GB, Tybjaerg-Hansen A, Benn M. Sex hormones and ischemic stroke: a prospective cohort study and meta-analyses. J Clin Endocrinol Metab. 2016;101:69-78.
