# Supplementary material for: Androgens In Men Study (AIMS): protocol for meta-analyses of individual participant data investigating associations of androgens with health outcomes in men
Source: BMJ Open. 2020 May 11;10(5):e034777. doi: 10.1136/bmjopen-2019-034777 (PMC7239545; doi:10.1136/bmjopen-2019-034777)
Supplement: Author's manuscript [file bmjopen-2019-034777.draft_revisions.pdf]

BMJ Open

BMJ Open is committed to open peer review. As part of this commitment we make the peer review history of every article we publish publicly available.

When an article is published we post the peer reviewers' comments and the authors' responses online. We also post the versions of the paper that were used during peer review. These are the versions that the peer review comments apply to.

The versions of the paper that follow are the versions that were submitted during the peer review process. They are not the versions of record or the final published versions. They should not be cited or distributed as the published version of this manuscript.

BMJ Open is an open access journal and the full, final, typeset and author-corrected version of record of the manuscript is available on our site with no access controls, subscription charges or pay-per-view fees (http://bmjopen.bmj.com).

If you have any questions on BMJ Open's open peer review process please email info.bmjopen@bmj.com

BMJ Open

Androgens In Men Study (AIMS): protocol for meta-analyses of individual participant data investigating associations of androgens with health outcomes in men.

Journal:	*BMJ Open*
Manuscript ID	bmjopen-2019-034777
Article Type:	Protocol
Date Submitted by the Author:	05-Oct-2019

Complete List of Authors:	Yeap, Bu; University of Western Australia, Medical School; Fiona Stanley Hospital, Department of Endocrinology and Diabetes<br>Marriott, Ross; University of Western Australia, School of Population and Global Health<br>Adams, Robert; Flinders University, Adelaide Institute for Sleep Health<br>Antonio, Leen; KU Leuven, Clinical and Experimental Endocrinology<br>Ballantyne, Christie; Baylor College of Medicine, Internal Medicine<br>Bhasin, Shalender; Harvard Medical School<br>Cawthon, PM; California Pacific Medical Center Research Institute, San Francisco Coordinating Center<br>Couper, David; University of North Carolina at Chapel Hill, Gillings School of Global Public Health<br>Dobs, Adrian; Johns Hopkins University, School of Medicine, Division of Endocrinology, Diabetes and Metabolism<br>Flicker, Leon; University of Western Australia, WA Centre for Health & Ageing<br>Karlsson, Magnus; Lund University, Department of Clinical Sciences and Orthopedic Surgery<br>Martin, Sean A.; The University of Adelaide, Freemasons Foundation Centre for Men's Health<br>Matsumoto, AM; VA Puget Sound Health Care System, Geriatric Research, Education and Clinical Center; University of Washington System, Medicine<br>Mellström, Dan ; University of Gothenburg, Centre for Bone and Arthritis Research at the Sahlgrenska Academy, Institute of Medicine<br>Norman, Paul; University of Western Australia, Medical School<br>Ohlsson , C; University of Gothenburg, Centre for Bone and Arthritis Research at the Sahlgrenska Academy, Institute of Medicine<br>Orwoll, Eric; Oregon Health & Science University<br>O'Neill, Terence; The University of Manchester & NIHR Manchester Biomedical Research Centre, Centre for Epidemiology Versus Arthritis, Faculty of Biology, Medicine and Health; Manchester Academic Health Science Centre, Manchester University NHS Foundation Trust<br>Shores, Molly; VA Puget Sound Health Care System; University of Washington, School of Medicine, Department of Psychiatry and Behavioral Sciences<br>Travison, Thomas; Beth Israel Deaconess Medical Center, Institute for Aging Research, Hebrew SeniorLife; Harvard Medical School<br>Vanderschueren, Dirk; Katholieke Universiteit Leuven, Department of

	Chronic Diseases, Metabolism and Ageing (CHROMETA), Laboratory of Clinical and Experimental Endocrinology<br>Wittert, Gary; The University of Adelaide, Freemasons Centre for Men's Health<br>Wu, Frederick; The University of Manchester, Division of Diabetes, Endocrinology and Gastroenterology<br>Murray, Kevin; University of Western Australia, School of Population and Global Health
Keywords:	Sex steroids & HRT < DIABETES & ENDOCRINOLOGY, EPIDEMIOLOGY, General endocrinology < DIABETES & ENDOCRINOLOGY

SCHOLARONE™
Manuscripts

I, the Submitting Author has the right to grant and does grant on behalf of all authors of the Work (as defined in the below author licence), an exclusive licence and/or a non-exclusive licence for contributions from authors who are: i) UK Crown employees; ii) where BMJ has agreed a CC-BY licence shall apply, and/or iii) in accordance with the terms applicable for US Federal Government officers or employees acting as part of their official duties; on a worldwide, perpetual, irrevocable, royalty-free basis to BMJ Publishing Group Ltd ("BMJ") its licensees and where the relevant Journal is co-owned by BMJ to the co-owners of the Journal, to publish the Work in this journal and any other BMJ products and to exploit all rights, as set out in our licence.

The Submitting Author accepts and understands that any supply made under these terms is made by BMJ to the Submitting Author unless you are acting as an employee on behalf of your employer or a postgraduate student of an affiliated institution which is paying any applicable article publishing charge ("APC") for Open Access articles. Where the Submitting Author wishes to make the Work available on an Open Access basis (and intends to pay the relevant APC), the terms of reuse of such Open Access shall be governed by a Creative Commons licence – details of these licences and which Creative Commons licence will apply to this Work are set out in our licence referred to above.

Other than as permitted in any relevant BMJ Author's Self Archiving Policies, I confirm this Work has not been accepted for publication elsewhere, is not being considered for publication elsewhere and does not duplicate material already published. I confirm all authors consent to publication of this Work and authorise the granting of this licence.

For peer review only

Androgens In Men Study (AIMS): protocol for meta-analyses of individual participant data investigating associations of androgens with health outcomes in men.

Corresponding author

Dr Bu B. Yeap MBBS, FRACP, PhD, Harry Perkins Institute of Medical Research, 11 Robin Warren Drive, Murdoch 6150, Western Australia, Australia.

Email: bu.yeap@uwa.edu.au; Telephone: +61 8 6151 1149

Authors:

Bu B. Yeap, Medical School, The University of Western Australia, Perth 6009, Australia.

Ross J. Marriott, School of Population and Global Health, The University of Western Australia, Perth 6009, Australia.

Robert J. Adams, Adelaide Institute for Sleep Health, Flinders University, Bedford Park 5042, Australia.

Leen Antonio, Clinical and Experimental Endocrinology, KU Leuven, Leuven, Belgium.

Christie M. Ballantyne, Internal Medicine, Baylor College of Medicine, Houston, Texas, USA.

Shalender Bhasin, Harvard Medical School, Boston, Massachusetts, USA.

Peggy M. Cawthon, San Francisco Coordinating Center, California Pacific Medical Center Research Institute, San Francisco, California, USA.

David J. Couper, Gillings School of Global Public Health, University of North Carolina at Chapel Hill, Chapel Hill, North Carolina, USA.

Adrian S. Dobs, School of Medicine, Division of Endocrinology, Diabetes and Metabolism, Johns Hopkins University, Baltimore, Maryland, USA.

For peer review only

Leon Flicker, WA Centre for Health & Ageing, The University of Western Australia, Perth 6009, Australia.

Magnus Karlsson, Department of Clinical Sciences and Orthopedic Surgery, Lund University, Lund, Sweden.

Sean A. Martin, Freemasons Foundation Centre for Men's Health, The University of Adelaide, Adelaide 5000, Australia.

Alvin M. Matsumoto, Geriatric Research, Education and Clinical Center, VA Puget Sound Health Care System, Seattle, Washington, USA.

Dan Mellström, Centre for Bone and Arthritis Research at the Sahlgrenska Academy, Institute of Medicine, University of Gothenburg, Goteburg, Sweden.

Paul E. Norman, Medical School, The University of Western Australia, Fremantle 6160, Australia.

Claes Ohlsson, Centre for Bone and Arthritis Research at the Sahlgrenska Academy, Institute of Medicine, University of Gothenburg, Goteburg, Sweden.

Eric S. Orwoll, Oregon Health & Science University, Portland, Oregon, USA.

Terence W. O'Neill, Centre for Epidemiology Versus Arthritis, Faculty of Biology, Medicine and Health, The University of Manchester & NIHR Biomedical Research Centre, Manchester, United Kingdom.

Molly M. Shores, VA Puget Sound Health Care System, Seattle, Washington, United States.

Thomas G. Travison, Institute for Aging Research, Hebrew SeniorLife, Beth Israel Deaconess Medical Center, Boston, Massachusetts, USA.

Dirk Vanderschueren, Department of Chronic Diseases, Metabolism and Ageing (CHROMETA), Laboratory of Clinical and Experimental Endocrinology, Katholieke Universiteit Leuven, Leuven, Flanders, Belgium.

For peer review only

For peer review only - http://bmjopen.bmj.com/site/about/guidelines.xhtml

Gary A. Wittert, Freemasons Foundation Centre for Men's Health, The University of Adelaide, Adelaide 5000, Australia.

Frederick C. W. Wu., Division of Diabetes, Endocrinology and Gastroenterology, The University of Manchester, Manchester, United Kingdom.

Kevin Murray, School of Population and Global Health, The University of Western Australia, Perth 6009, Australia.

For peer review only

Word count, excluding title page, abstract, references, acknowledgements, contributions, figures and tables:

3,374 words.

For peer review only - http://bmjopen.bmj.com/site/about/guidelines.xhtml

ABSTRACT

Introduction

This study aims to clarify the role(s) of endogenous sex hormones to influence health outcomes in men, specifically to define the associations of plasma testosterone with incidence of cardiovascular events, cancer, dementia and mortality risk, and to identify factors predicting testosterone concentrations. Data will be accrued from at least three Australian, two European, and four North American population-based cohorts involving approximately 20,000 men.

Methods and analysis

Eligible studies include prospective cohort studies with baseline testosterone concentrations measured using mass spectrometry and five years of follow-up data on incident cardiovascular events, mortality, cancer diagnoses or deaths, new onset dementia, or decline in cognitive function recorded. Data for men, who were not taking androgens or drugs suppressing testosterone production, metabolism, or action; and had no prior orchidectomy, are eligible. The date range of bibliographic searches will not be constrained, and aggregate level data will be sought where individual participant data (IPD) is not available. One-stage IPD random-effects meta-analyses will be performed, using linear mixed models, generalised linear mixed models and either stratified or frailty-augmented Cox regression models. Heterogeneity in estimates from different studies will be quantified and bias investigated using funnel plots. Effect size estimates will be presented in forest plots and non-negligible heterogeneity and bias investigated using subgroup or meta-regression analyses.

Ethics and dissemination

Ethics approvals obtained for each of the participating cohorts state that participants have consented to have their data collected and used for research purposes. There are four planned research articles, with each involving a separate set of IPD meta-analyses.

For peer review only

For peer review only - http://bmjopen.bmj.com/site/about/guidelines.xhtml

Registration [Systematic Review Protocol ID 139668 submitted to Prospero on 23 July 2019 and is presently being assessed].

STRENGTHS AND LIMITATIONS OF THIS STUDY

- A large international collaboration combining data from multiple cohorts will enable definitive analyses whose results possess global applicability. Investigators from at least three large Australian, two European, and four North American cohorts (each with N > 1,000 participants), which have IPD-level data available, have agreed to collaborate and confirmed availability of suitable IPD-level data, provisional on approvals from their local IRBs. Aggregate level data will be sought in the event IPD-level data are not available.
- Harmonization will be required for some variables (e.g., physical activity, alcohol consumption) that are recorded differently by the component studies.
- Analyses will allow reference ranges for testosterone in men across ages and geographical locations to be refined to inform recommendations for clinical practice.

For peer review only

INTRODUCTION

As men grow older, testosterone production and circulating concentrations of testosterone decline while comorbidities accumulate.[1-4] Older men, even those in very good health, have lower circulating testosterone concentrations compared with healthy young men.[5, 6] Although results have been inconsistent, an increasing number of studies have reported associations of low endogenous testosterone concentration with poorer health outcomes, especially in older men. For instance, studies that have used liquid chromatography tandem mass spectrometry, widely regarded as the reference method for the measurement of total testosterone concentrations,[7] have reported associations of lower endogenous testosterone concentrations with: (i) cardiovascular disease and all-cause mortality in some cases[8-15] but not others[8, 16-20]; (ii) some cancers but not others[21, 22]; and (iii) dementia[23, 24] but not laboratory measures of cognitive function.[25] Therefore it remains unclear whether testosterone is a biomarker of ill-health or a causal factor for diseases of ageing.

Currently, testosterone treatment is recommended for men who have symptoms and signs of androgen deficiency and low testosterone concentrations, due to disease of the hypothalamus, pituitary or testes (organic or pathological hypogonadism).[26-29] Randomised controlled trials of testosterone treatment in older men with low-normal testosterone concentrations without organic hypogonadism have shown modest benefits on sexual function, anaemia, self-reported physical function and mobility, and volumetric bone density, but not on some objective measures of cognition over 12 to 36 months.[30-34] The effect of testosterone on major adverse cardiovascular events remains unclear.[35, 36] However, the selection criteria of these trials was such that the screening to enrolment ratio was 65:1, a highly selected population of older men.[32, 33, 36] Importantly, the trials were neither large enough nor long enough to determine the effects of testosterone on major adverse cardiovascular events,

For peer review only

For peer review only - http://bmjopen.bmj.com/site/about/guidelines.xhtml

development of dementia, bone fractures, and mortality.[36] Therefore, a meta-analysis of data from prospective cohort studies, with extended follow-up periods, provides opportunities for better understanding of the temporal profiles of the postulated associations between endogenous testosterone concentration and incident health outcomes. Meta-analyses of Individual Participant Data (IPD) are generally preferable to meta-analyses of aggregate data in that they typically have higher statistical power and provide scope to control for important confounders and risk factors.[37, 38] Furthermore, one-stage IPD meta-analyses are preferable to two-stage approaches because the former uses an exact likelihood to directly model the distribution of IPD, offers the convenience of using a standard set of diagnostic tools to assess model fit, and can arguably provide greater flexibility, in terms of options for statistical modelling.[39, 40]

The Androgens In Men Study (AIMS), an international collaboration of prospective cohort studies, will examine the associations of sex hormones with health outcomes that are major sources of morbidity and mortality in middle-aged and older men. The group will perform a series of IPD meta-analyses to clarify the influence of sex hormone exposures on major health outcomes, including heart attack, stroke and cardiovascular deaths, cancer, new-onset dementia, and all-cause mortality, as well as provide information on social, demographic and behavioural factors that are associated with endogenous testosterone concentrations. This work will characterise robustly the associations of androgens with health outcomes in men in general over and above the study-specific estimates, thus clarifying the role of androgens as biomarkers for, or causal contributors to, men's health.

For peer review only - http://bmjopen.bmj.com/site/about/guidelines.xhtml

Objectives

The Androgens In Men Study (AIMS) will establish an international collaboration of existing cohort studies to clarify the relation of endogenous sex hormones with major health outcomes in men. PEO (Population, Exposure, Outcomes) characteristics include: adult men in the general community; an exposure of endogenous circulating sex hormone concentration, primarily testosterone, as the principal male sex hormone or androgen; and a prospective cohort study type, with incident health outcomes including incidence of cardiovascular disease events, mortality, cancers, and dementia. The specific objectives of the AIMS study are: to investigate associations between variables representing social, demographic, and behavioural factors with the measured concentration of androgens in the blood of men (Analysis 1); to examine the associations between androgen concentrations and subsequent incidence of cardiovascular events, cardiovascular deaths, and all-cause mortality in men (Analysis 2); to examine the associations between androgen concentrations and subsequent mortality from (and, if available, diagnoses of) common cancers in men (Analysis 3); to examine the associations between androgen concentrations and cognitive impairment and incident dementia in men (Analysis 4). Analysis 2 will evaluate myocardial infarction, stroke and heart failure, deaths due to cardiovascular disease, and the composite endpoint of major adverse cardiovascular events (MACE) comprising non-fatal myocardial infarction, non-fatal stroke, and deaths due to cardiovascular disease. Analysis 3 will evaluate outcomes of deaths due to and diagnoses of colorectal cancer, lung cancer and prostate cancer.

For peer review only - http://bmjopen.bmj.com/site/about/guidelines.xhtml

METHODS AND ANALYSIS

IPD meta-analyses will be conducted to understand the associations between testosterone and a range of associated major health outcomes in men. IPD meta-analyses have been selected as the most suitable approach because: (i) the required aggregate data (AD) are not available from each of the cohorts in published literature; (ii) IPD meta-analyses typically have higher statistical power than AD meta-analyses and provide scope for controlling for important confounders and risk factors.[37, 38] Where possible, methods will adhere to the guidelines for Preferred Reporting Items for Systematic Reviews and Meta-Analyses of IPD data (PRISMA-IPD; completed checklist is provided in Supplementary Material) and the Meta-analysis Of Observational Studies in Epidemiology (MOOSE).[41-43]

Studies selected for inclusion in IPD meta-analyses

Studies will be identified from a systematic review using online search tools for mainstream published (MEDLINE, EMBASE) and grey literature (OpenGrey, Mednar) studies (Prospero application for registration No. 139668 submitted 23 July 2019). Eligible studies include prospective cohort studies with plasma or serum testosterone concentrations measured using mass spectrometry with at least five years of follow-up data, with incident cardiovascular, cancer, mortality, dementia, or cognitive events recorded (see Supplementary Table S1 for an example of search criteria to be used). The search strategy selects for articles based on words or Medical Subject Headings (MeSH) terms matching the relevant exposure (example steps 1-3), outcomes (example steps 4-10), and study type (example steps 14-15), then, depending on the search tool, filters down to more relevant studies, with exclusions of clinical trials and of studies on non-humans (example steps 18-27), with no date range restrictions. Ahead of the systematic review, nine eligible studies (cohorts) had expressed interest to collaborate: three from Australia (Busselton Health Study BHS, Health In Men Study HIMS, Men

For peer review only - http://bmjopen.bmj.com/site/about/guidelines.xhtml

Androgen Inflammation Lifestyle Environment and Stress study MAILES); two from Europe (European Male Ageing Study EMAS, Osteoporotic Fractures in Men Study MrOS - Sweden); and four from the USA (Atherosclerosis Risk in Communities study ARIC, Cardiovascular Health Study CHS, The Framingham Heart Study FHS, Osteoporotic Fractures in Men Study MrOS - USA). Investigators from eight of these studies have confirmed availability of suitable IPD-level data, provisional on approvals from their respective Publication and Steering Committees. If it is not possible to obtain IPD-level data from selected studies, we will request suitable AD-level data.

Data provision, merging, harmonisation, and storage

The project manager will liaise directly with the nominated contact person for each cohort study to identify, specifically, which variables and set of observations will be suitable to request. A data request will then be submitted to the data custodian for each study. Requested variables will be labelled as either “highly desirable” or “only if available”, in order to prioritise efforts in obtaining the key variables for analyses and to acknowledge the differing availability of variables among studies. A list of variable names, definitions, and attributes, including numbers of rows and columns in each data file(s) will also be requested to be provided separately. Methods for ascertaining outcome and comorbidity status will be requested, which can be used to indicate the relative quality of diagnostic information (e.g. ICD-coded diagnoses from hospital inpatient admissions versus self-report information). File transfer will be achieved via encrypted file transfer (or other sufficiently secure method).

Once each dataset has been received by the project manager, the original file(s) will be saved and date-stamped in a secure central repository. All subsequent manipulations will be completed using copies of the original file, with syntax saved as script files. Variable

For peer review only

For peer review only - http://bmjopen.bmj.com/site/about/guidelines.xhtml

definitions will be checked, variables inspected for missing values, and variable properties and value ranges assessed to identify possible outliers. A table of summary statistics will be calculated and, where possible, analyses run and compared with published values to check for data consistency. The nature of any discrepancies identified from these checks will need to be understood, and possibly resolved, prior to proceeding with the meta-analyses.[42, 44, 45] Missing data will be suitably imputed.[45, 46]

Prior to the merging of datasets, a variable to identify each source study will be appended as the new first column. Variable formats will be checked and corrected for consistency and anonymised participant identifier codes for uniqueness across all studies. Harmonization will be required for some variables (e.g., physical activity, alcohol consumption) that are recorded differently by the different component studies. Where possible, AD datasets will be used to reconstruct IPD-level data (that is, partially-reconstructed IPD) prior to merging.[47-49] Should this not be possible, the IPD-level data will be aggregated and summary estimates made with and without AD-level data as sensitivity analyses. It is also possible that some studies might have outcomes available for some analyses, but not others; in these cases, we will preferentially use IPD-level data when available but also seek to use AD-level data from those other studies when available. There is no requirement to use IPD-level data from the same studies across all analyses.

All IPD-level data will be accessible to only the approved staff from a secure on-site facility, from rooms that are kept locked when unattended, and with remote access not permitted. IPD-level data are not to be printed in hard copy and will only be presented at aggregate level. Data analysed for this study will be retained for five years after the last of all proposed analyses are published and will then be destroyed. A post-analysis retention period is required

For peer review only - http://bmjopen.bmj.com/site/about/guidelines.xhtml
For peer review only

to enable publication and possible scrutiny of findings. Disposal will be carried out according to best practice.

Data items

The full list of generic variables to include in meta-analyses are presented in Table 1. Analysis 1 will model relationships of androgen concentrations, as measured in serum or plasma samples (dependent variable), with key demographic variables and risk factors for disease (predictor variables). Time-to-event variables obtained from follow-up data will be analysed in Analysis 2 and 3 (outcomes), and their associations with androgen concentrations (focal predictor) and other potential confounders and risk factors (predictor variables). Records of dementia diagnoses (physician or otherwise categorised) and relative cognitive function (for example, from test scores) will be analysed in Analysis 4, and their associations with androgen concentrations and other potential confounders and risk factors. Androgen variables will include measurements of total testosterone and, if available, of dihydrotestosterone, estradiol, luteinising hormone (LH), and Sex Hormone Binding Globulin (SHBG). For exploratory analyses, free testosterone, the amount in the circulation which is not protein-bound, will be calculated from measured total testosterone and SHBG.[50] Covariates were selected to include those used in previous studies, as well as those that are typically recorded. The full list of proposed variables to include in the meta-analyses are presented in Table 1.

Statistical analysis

Since the datasets to be analysed are sampled from different populations, a random-effects meta-analysis is appropriate, as it acknowledges that effects will vary among studies due to

For peer review only

For peer review only - http://bmjopen.bmj.com/site/about/guidelines.xhtml

differences in local factors.[51] One-stage IPD meta-analyses will be performed. Each IPD meta-analysis will involve fitting a model with study estimated as either a fixed or random term, to account for related observations within studies, and androgen modelled as random slopes (when modelled as a continuous predictor) or intercepts (when modelled as a categorical predictor), to harmonised, merged data from all cohorts. The underlying statistical model and estimates of effect size will be specific to each of the proposed analyses and are outlined as follows.

Analysis 1: Factors associated with testosterone concentrations in men and characterisation of reference ranges.

Linear Mixed Models (LMMs) or Generalised Linear Mixed Models (GLMMs) will be used to model the relation between the predictors (independent variables) and each hormonal variable (testosterone, dihydrotestosterone, estradiol, LH, SHBG) as five separate IPD meta-analyses. Suspected non-linear relationships, at the scale of the linear predictor, will be investigated and modelled appropriately (e.g., splines with pre-set knot locations and linear boundary constraints). Measures of effect size may include (but are not limited to): η^2,[52, 53] Pearson's r, standardised mean difference to the reference level (categorical predictors), standardised difference for an increase in one standard deviation (continuous predictors). In the case of non-linear relations, we will graphically describe the relationship with comparisons made with appropriate reference points. Reference ranges will be derived based on the distributions of testosterone in healthy men.

For peer review only - http://bmjopen.bmj.com/site/about/guidelines.xhtml

Analysis 2: Associations between testosterone concentrations and subsequent incidence of cardiovascular events, cardiovascular deaths, and all-cause mortality.

Cox proportional hazards models will be used to assess the effect of androgen level on the incident risk of each outcome, with separate IPD meta-analyses conducted for each outcome (myocardial infarction, stroke, heart failure, deaths due to cardiovascular disease, and MACE) and each hormonal variable (testosterone, dihydrotestosterone, estradiol, LH, SHBG). Component study will be modelled either as a stratified variable[39] or as a random term, and androgen as a random term, using frailty models, which are a class of survival models that incorporate random effects.[54-56] Participants with prevalent cardiovascular disease at baseline will be excluded. The length of follow-up will also be standardised among studies in order to maximise data from all datasets, whilst minimising the prospect for variable lengths-to-follow-up among studies introducing additional heterogeneity into results.[46]

Multivariable versions of each of these models will also be fitted, with additional predictors for potential confounders and risk factors included (Table 1). Non-linear associations for continuous variables will be modelled using natural splines with pre-specified knots and linear boundary constraints. The (standardised) measure of effect size used will be the hazard ratio. Subgroup analyses will be conducted separately for each of three specific types of cardiovascular disease (outcomes): myocardial infarction, stroke and heart failure.

Analysis 3: Association between testosterone level and subsequent incidence of cancer.

Cox proportional hazards models will be used to assess the effect of androgen concentrations on the incident risk of cancer deaths and, if available, of cancer diagnoses. IPD meta-analyses

For peer review only - http://bmjopen.bmj.com/site/about/guidelines.xhtml

separately conducted for each of these outcomes and each hormonal variable as focal predictor (testosterone, dihydrotestoterone, estradiol, LH, SHBG). Component study will be modelled either as a stratified variable[39] or as a random term, and androgen as a random term, using frailty models.[54-56] Participants with prevalent cancer diagnosis at baseline will be excluded. The length of follow-up will be standardised among studies in order to maximise data from all datasets, whilst minimising the prospect for variable lengths-to-follow-up among studies introducing additional heterogeneity into results.[46]

Multivariable versions of each of these models will also be fitted, with additional predictors for potential confounders and risk factors included (Table 1). Non-linear associations for continuous variables will be modelled using natural splines with pre-specified knots and linear boundary constraints. The (standardised) measure of effect size used will be the hazard ratio. Subgroup analyses will be conducted separately for each of three common types of cancers in men (outcomes): colorectal cancer, lung cancer, prostate cancer. For these analyses, men with the relevant cancer type at baseline will be excluded from that specific analysis. Thus men with prevalent colorectal cancer (but not other cancer types) will be excluded in the analysis of incident colorectal cancer; similarly for the analyses of incident lung and prostate cancer, men with lung or prostate cancer at baseline will be excluded.

Analysis 4: Associations of androgen levels with cognitive impairment and incident dementia in men.

LMMs and GLMMs will be used to model the association of androgen concentrations with cognitive impairment (cross-sectional analyses of baseline data). Cox proportional hazards models will be used to assess the effect of androgen concentrations on the incident risk of dementia. Men with prevalent dementia will be excluded from this analysis. We will also ask

For peer review only

For peer review only - http://bmjopen.bmj.com/site/about/guidelines.xhtml

for follow-up cognition test scores, and if available, will run an analysis of changes in cognition test scores from baseline as an outcome. Separate IPD meta-analyses will be conducted for each hormonal variable as a focal predictor (testosterone, dihydrotestosterone, estradiol, LH, SHBG). Component study will be modelled as a fixed or random intercept term in LMMs and GLMMs and as a stratified factor or frailty model random term in Cox regressions. Multivariable versions of each of these models will also be fitted, with additional predictors for potential confounders and risk factors included (Table 1). Suspected non-linear relationships, at the scale of the linear predictor, will be investigated and modelled using splines with pre-specified knots and boundary constraints. Measures of effect size may include: η^2, Pearson's r, standardised mean difference to the reference level (categorical predictors), standardised difference for an increase in one standard deviation (continuous predictors), odds ratio for LMMs and GLMMs and hazard ratio for Cox regressions.

Throughout all analyses, contour-enhanced funnel plots will be constructed to visually assess patterns in estimates of effect sizes and precision among studies, to investigate heterogeneity and possible meta-biases.[57-59] The relative amount of heterogeneity will be estimated (e.g., using I^2) and forest plots presented. Subgroup or meta-regression analyses may be conducted if pronounced heterogeneity is estimated.[51]

Patient and public involvement

The level and type of participant/patient and public involvement will pertain to each respective component study used for the meta-analyses, with additional studies to be identified from systematic review. Accordingly, we refer the reader to respective published journal articles for each component study for further details.[60-67]

For peer review only - http://bmjopen.bmj.com/site/about/guidelines.xhtml

ETHICS AND DISSEMINATION

Ethics approvals obtained for each of the participating cohorts state that participants have consented to have their data collected and used for research purposes. Furthermore, there are no expected harms or risks to participants, as data have already been collected within each individual cohort, under existing ethics approvals. De-identified data will be collated and analysed, with no new procedures planned for participants. The AIMS study has been assessed as exempt from ethics review by the Human Research Ethics Office at the University of Western Australia (file reference number RA/4/20/5014).

DISCUSSION

Although several published meta-analyses have investigated associations of endogenous testosterone with health outcomes in men,[68-79] none have conducted IPD meta-analyses of health outcomes as planned for this study. A previous IPD meta-analysis focussed on the outcome of metabolic syndrome.[71] Results from this study will improve upon previously published estimates from individual studies, in terms of the generalisability of findings. Estimates from the IPD meta-analyses are also likely to be more reliable than those published from conventional meta-analyses because they typically have higher statistical power and provide scope for controlling for important confounders and risk factors.[37, 38] Uncertainty will undoubtedly remain due to the possibility of confounding influences of unadjusted effects. However, unlike a randomised controlled trial, this study avoids the need to subject individuals to interventions, and provides more comprehensive characterisation of temporal relationships between baseline androgen concentrations and a range of key health outcomes.[80]

For peer review only - http://bmjopen.bmj.com/site/about/guidelines.xhtml

Accordingly, it is hoped that the AIMS collaboration will ultimately complement the research efforts and outputs from multiple prospective cohort studies by drawing upon the collective body of evidence to clarify the role of endogenous sex hormone levels on major health outcomes in men. It is possible that this work might also elucidate new understanding, arising from improved scope for fitting more complex models due to increased statistical power, or from patterns detected in subgroup or meta-regression analyses. Clinically, research outputs will be used to identify the scope and optimal recruitment criteria for future trials of testosterone therapy. These data will also allow reference ranges for testosterone in men across ages and geographical locations to be refined, to inform recommendations for clinical practice more generally.

For peer review only

CONCLUSION

This new collaboration (the AIMS study) and series of IPD-meta analyses described here will complement previous research efforts and outputs from a number of prospective cohort studies for describing associations between endogenous androgen concentrations and health outcomes in men. Research outputs will serve to progress the collective body of knowledge and clarify the importance of testosterone for male health more generally, with several key implications for clinical practice, including prevention (of testosterone decline), personalised medicine and testosterone therapy.

Acknowledgements

We thank Östen Ljunggren of the Department of Medical Sciences, Uppsala University and Liesbeth Vandenput of the Centre for Bone and Arthritis Research at the Sahlgrenska Academy Institute of Medicine, University of Gothenburg, Mary Lou Biggs, Terena Solomons, Jos Tournoy, Mark Divitni, Matthew Knuiman, John Acres, the staff and

For peer review only - http://bmjopen.bmj.com/site/about/guidelines.xhtml

participants of the ARIC study, the Busselton Population Medical Research Foundation for access to the data, the Cardiovascular Health Study, the EMAS study, the Health In Men Study, the MAILES study, the MrOS Study, for their important contributions.

Collaborators

The Androgens In Men Study: (1) Busselton Health Study: Busselton Population Medical Research Institute (Inc.), Department of Pulmonary Physiology and Sleep Medicine, Sir Charles Gairdner Hospital, Nedlands WA 6009, Australia (2) Health In Men Study (HIMS): Western Australian Centre for Health and Ageing, University of Western Australia, Level 6 Medical Research Foundation Building, Royal Perth Hospital, Rear 50 Murray Street, Perth, Western Australia 6000, Australia. (3) Men Androgen Inflammation Lifestyle Environment and Stress (MAILES) study: Freemasons Foundation Centre for Men's Health, University of Adelaide, 254 North Tce, Adelaide, Australia. (4) European Male Ageing Study (EMAS): The University of Manchester, Stopford Building, Oxford Road, Manchester, M13 9PT, UK. (5) Osteoporotic Fractures in Men (MrOS) Study Sweden: Department of Clinical Sciences and Orthopedic Surgery, Lund University, Skåne University Hospital, Malmö, Sweden; MrOS Study USA: California Pacific Medical Center Research Institute, San Francisco Coordinating Center, 550 16th street, San Francisco, CA 94143. (6) Atherosclerosis Risk in Communities study (ARIC): School of Public Health, U. of North Carolina, Suite 2013, NCNB Plaza, 137 E. Franklin St, Chapel Hill, NC 27514. (7) Cardiovascular Health Study: National Heart, Lung, and Blood Institute, Bethesda, MD. (8) The Framingham Heart Study, Boston University School of Medicine, 72 E. Concord Street, B-601, Boston, MA 02118.

For peer review only

Authors' contributions

BBY, KM, LA, SB, ASD, AMM, CO, EO, TWO, DV, GAW, contributed to the study concept and design. BBY, KM, RJM prepared the initial draft of the manuscript, including literature review, list of variables and statistical methods. All authors were involved in subsequent revisions to the protocol and manuscript, and approved this submission.

Funding

AIMS is funded by a Western Australian Health Translation Network (WAHTN) Medical Research Future Fund Rapid Applied Translation Grant (2018). The ARIC study is funded by has been funded in whole or in part with Federal funds from the National Heart, Lung, and Blood Institute, National Institutes of Health, Department of Health and Human Services, under Contract nos. (HHSN268201700001I, HHSN268201700002I, HHSN268201700003I, HHSN268201700005I, HHSN268201700004I) and Dr. Ballantyne received NIH grant support through R01HL134320 The 1994/1995 Busselton Health Survey was supported by the Western Australian Health Promotion Foundation. The Cardiovascular Health Study was supported by contracts HHSN268201200036C, HHSN268200800007C, HHSN268201800001C, N01HC55222, N01HC85079, N01HC85080, N01HC85081, N01HC85082, N01HC85083, N01HC85086, and grants U01HL080295 and U01HL130114 from the National Heart, Lung, and Blood Institute (NHLBI), with additional contribution from the National Institute of Neurological Disorders and Stroke (NINDS). Additional support was provided by R01AG023629 from the National Institute on Aging (NIA). A full list of principal CHS investigators and institutions can be found at CHS-NHLBI.org. The EMAS study is funded by The Commission of the European Communities Fifth Framework Programme "Quality of Life and Management of Living Resources" Grant No. QLK6-CT-2001-00 258 and supported by Arthritis Research UK. The Framingham Heart Study of the

For peer review only

For peer review only - http://bmjopen.bmj.com/site/about/guidelines.xhtml

National Heart Lung and Blood Institute of the National Institutes of Health and Boston University School of Medicine has been funded in whole or in part with Federal funds from the National Heart, Lung, and Blood Institute, National Institutes of Health, Department of Health and Human Services, under Contract No. 75N92019D00031. The HIMS study is funded by project grants from the National Health and Medical Research Council of Australia. The MAILES study is funded by a project grant from the National Health and Medical Research Council of Australia (627227). The MrOS Sweden study is funded by The Swedish Research Council and Swedish ALF funding. The Osteoporotic Fractures in Men (MrOS) Study is supported by National Institutes of Health funding. The following institutes provide support: the National Institute on Aging (NIA), the National Institute of Arthritis and Musculoskeletal and Skin Diseases (NIAMS), the National Center for Advancing Translational Sciences (NCATS), and NIH Roadmap for Medical Research under the following grant numbers: U01 AG027810, U01 AG042124, U01 AG042139, U01 AG042140, U01 AG042143, U01 AG042145, U01 AG042168, U01 AR066160, and UL1 TR000128.

Disclaimer

The funders played no role in the study design, data collection, data analysis or interpretation, writing of the manuscript or the decision to submit the article for publication. All authors are accountable for all aspects of the work in ensuring that questions related to the accuracy or integrity of any part of the work are appropriately investigated and resolved. The content is solely the responsibility of the authors and does not necessarily represent the official views of the National Institutes of Health.

Competing interests

None declared.

For peer review only - http://bmjopen.bmj.com/site/about/guidelines.xhtml

For peer review only

Patient consent

Each of the component studies have addressed participant/patient consent permissions; please refer to respective component studies for details.[60-67]

Ethics approval

The AIMS study has been assessed as exempt from ethics review by the Human Ethics office at the University of Western Australia (file reference number RA/4/20/5014). Each of the component studies had obtained ethics approvals; please refer to respective component studies for details.[60-67]

Provenance and peer review

Not commissioned; externally peer reviewed.

For peer review only - http://bmjopen.bmj.com/site/about/guidelines.xhtml

References cited

1. Handelsman DJ, Yeap BB, Flicker L, et al. Age-specific population centiles for androgen status in men. *Eur J Endocrinol* 2015;173(6):809-17. doi: 10.1530/EJE-15-0380
2. Hsu B, Cumming RG, Hirani V, et al. Temporal trend in androgen status and androgen-sensitive outcomes in older men. *J Clin Endocrinol Metab* 2016;101(4):1836-46. doi: 10.1210/jc.2015-3810
3. Shi Z, Araujo AB, Martin S, et al. Longitudinal changes in testosterone over five years in community-dwelling men. *J Clin Endocrinol Metab* 2013;98(8):3289-97. doi: 10.1210/jc.2012-3842
4. Yeap BB, Manning L, Chubb SAP, et al. Progressive impairment of testicular endocrine function in ageing men: testosterone and dihydrotestosterone decrease, and luteinizing hormone increases, in men transitioning from the 8th to 9th decades of life. *Clin Endocrinol* 2018;88:88-95. doi: 10.1111/cen.13484
5. Sikaris K, McLachlan RI, Kazlauskas R, et al. Reproductive hormone reference intervals for healthy fertile young men: evaluation of automated platform assays. *J Clin Endocrinol Metab* 2005;90(11):5928-36. doi: 10.1210/jc.2005-0962
6. Yeap BB, Alfonso H, Chubb SAP, et al. Reference ranges and determinants of testosterone, dihydrotestosterone, and estradiol levels measured using liquid chromatography-tandem mass spectrometry in a population-based cohort of older men. *J Clin Endocrinol Metab* 2012;97(11):4030-39. doi: 10.1210/jc.2012-2265
7. Handelsman DJ, Wartofsky L. Requirement for Mass Spectrometry Sex Steroid Assays in the Journal of Clinical Endocrinology and Metabolism. *J Clin Endocrinol Metab* 2013;98:3971-73. doi: 10.1210/jc.2013-3375
8. Hsu B, Cumming RG, Naganathan V, et al. Temporal Changes in Androgens and Estrogens Are Associated With All-Cause and Cause-Specific Mortality in Older Men. *J Clin Endocrinol Metab* 2016;101:2201-10. doi: 10.1210/jc.2016-1025
9. Ohlsson C, Barrett-Connor E, Bhasin S, et al. High Serum Testosterone Is Associated With Reduced Risk of Cardiovascular Events in Elderly Men. *J Am Coll Cardiol* 2011;58:1674-81. doi: 10.1016/j.jacc.2011.07.019
10. Pye SR, Huhtaniemi IT, Finn JD, et al. Late-Onset Hypogonadism and Mortality in Aging Men. *J Clin Endocrinol Metab* 2014;99:1357-66. doi: 10.1210/jc.2013-2052
11. Tivesten A, Mellstrom D, Jutberger H, et al. Low serum testosterone and high serum estradiol associate with lower extremity peripheral arterial disease in elderly men. *J Am Coll Cardiol* 2007;50:1070-76. doi: 10.1016/j.jacc.2007.04.088
12. Tivesten A, Vandenput L, Labrie F, et al. Low serum testosterone and estradiol predict mortality in elderly men. *J Clin Endocrinol Metab* 2009;94:2482-88. doi: 10.1210/jc.2008-2650
13. Yeap BB, Alfonso H, Chubb SAP, et al. In older men, higher plasma testosterone or dihydrotestosterone is an independent predictor for reduced incidence of stroke but not myocardial infarction. *J Clin Endocrinol Metab* 2014;99(12):4565-73. doi: 10.1210/jc.2014-2664
14. Yeap BB, Hyde Z, Almeida OP, et al. Lower testosterone levels predict incident stroke and transient ischemic attack in older men. *J Clin Endocrinol Metab* 2009;94(7):2353-59. doi: 10.1210/jc.2008-2416
15. Yeap BB, Knuiman MW, Divitni ML, et al. Differential associations of testosterone, dihydrotestosterone and oestradiol with physical, metabolic and health-related factors in community-dwelling men aged 17–97 years from the Busselton Health Survey. *Clin Endocrinol* 2014;81:100-08.
16. Chan YX, Knuiman MW, Hung J, et al. Neutral associations of testosterone, dihydrotestosterone and estradiol with fatal and non-fatal cardiovascular events, and mortality in men aged 17–97 years. *Clin Endocrinol* 2016;85:575-82. doi: 10.1111/cen.13089

For peer review only - http://bmjopen.bmj.com/site/about/guidelines.xhtml

17. Shores MM, Arnold AM, Biggs ML, et al. Testosterone and dihydrotestosterone and incident ischaemic stroke in men in the Cardiovascular Health Study. *Clin Endocrinol* 2014;81:746-53. doi: 10.1111/cen.12452
18. Shores MM, Biggs ML, Arnold AM, et al. Testosterone, Dihydrotestosterone, and Incident Cardiovascular Disease and Mortality in the Cardiovascular Health Study. *J Clin Endocrinol Metab* 2014;99:2061-68. doi: 10.1210/jc.2013-3576
19. Srinath R, Golden SH, Carson KA, et al. Endogenous Testosterone and its Relationship to Preclinical and Clinical Measures of Cardiovascular Disease in the Atherosclerosis Risk in Communities Study. *J Clin Endocrinol Metab* 2015;100:1602-08. doi: 10.1210/jc.2014-3934
20. Srinath R, Gottesman RF, Golden SH, et al. Association Between Endogenous Testosterone and Cerebrovascular Disease in the ARIC Study (Atherosclerosis Risk in Communities). *Stroke* 2016;47:2682-88. doi: 10.1161/STROKEAHA.116.014088
21. Chan YX, Knuiman MW, Divitni ML, et al. Lower circulating androgens are associated with overall cancer risk and prostate cancer risk in men aged 25-84 years from the Busselton Health Study. *Horm Cancer* 2018;9:391-98. doi: 10.1007/s12672-018-0346-5
22. Daniels NA, Nielson CM, Hoffman AR, et al. Sex hormones and the risk of incident prostate cancer. *Urology* 2010;76:1034-14. doi: 10.1016/j.urology.2010.01.086
23. Ford AH, Yeap BB, Flicker L, et al. Sex hormones and incident dementia in older men: The health in men study. *Psychoneuroendocrinol* 2018;98:139-47. doi: 10.1016/j.psyneuen.2018.08.013
24. Ford AH, Yeap BB, Flicker L, et al. Prospective longitudinal study of testosterone and incident depression in older men: The Health In Men Study. *Psychoneuroendocrinology* 2016;64:57-65. doi: 10.1016/j.psyneuen.2015.11.012
25. LeBlanc ES, Wang PY, Janowsky JS, et al. Association between sex steroids and cognition in elderly men. *Clin Endocrinol* 2010;72:393-403. doi: 10.1111/j.1365-2265.2009.03692.x
26. Yeap BB, Wu FCW. Clinical practice update on testosterone therapy for male hypogonadism: contrasting perspectives to optimize care. *Clin Endocrinol* 2019;90:56-65. doi: 10.1111/cen.13888
27. Yeap BB, Grossmann M, McLachlan RI, et al. Endocrine Society of Australia position statement on male hypogonadism (part 2): treatment and therapeutic considerations. *Med J Aust* 2016;205:228-31. doi: 10.5694/mja16.00448
28. Yeap BB, Grossmann M, McLachlan RI, et al. Endocrine Society of Australia position statement on male hypogonadsm (part 1): assessment and indications for testosterone therapy. *Med J Aust* 2016;205:173-78. doi: 10.5694/mja16.00393
29. Bhasin S, Brito JP, Cunningham GR, et al. Testosterone therapy in men with hypogonadism: an Endocrine Society* Clinical Practice Guideline. *J Clin Endocrinol Metab* 2018;103(5):1715-44. doi: 10.1210/jc.2018-00229
30. Resnick SM, Matsumoto AM, Stephens-Shields AJ, et al. Testosterone treatment and cognitive function in older men with low testosterone and age-associated memory impairment. *JAMA* 2017;317:717-27. doi: 10.1001/jama.2016.21044
31. Roy CN, Snyder PJ, Stephens-Shields AJ, et al. Association of testosterone levels with anemia in older men: A controlled clinical trial. *JAMA Intern Med* 2017;177:480-90. doi: 10.1001/jamainternmed.2016.9540
32. Snyder PJ, Bhasin S, Cunningham GR, et al. Effects of testosterone treatment in older men. *N Engl J Med* 2016;374:611-24. doi: 10.1056/NEJMoa1506119
33. Snyder PJ, Bhasin S, Cunningham GR, et al. Lessons from the testosterone trials. *Endocr Rev* 2018;39:369-86. doi: 10.1210/er.2017-00234
34. Snyder PJ, Kopperdahl DL, Stephens-Shields AJ, et al. Effect of testosterone treatment on volumetric bone density and strength in older men with low testosterone: A controlled clinical trial. *JAMA Intern Med* 2017;177:471-79. doi: 10.1001/jamainternmed.2016.9539
35. Budoff M, Ellenberg SS, Lewis CE, et al. Testosterone treatment and coronary artery plaque volume in older men with low testosterone. *JAMA* 2017;317:708-16. doi: 10.1001/jama.2016.21043

For peer review only

For peer review only - http://bmjopen.bmj.com/site/about/guidelines.xhtml

36. Yeap BB, Page ST, Grossmann M. Testosterone treatment in older men: clinical implications and unresolved questions from the Testosterone Trials. *Lancet Diabetes Endocrinol* 2018;6:659-72. doi: 10.1016/S2213-8587(17)30416-3
37. Riley RD, Lambert PC, Abo-Zaid G. Meta-analysis of individual participant data: rationale, conduct, and reporting. *BMJ* 2010; 340.
38. Stewart LA, Parmar MKB. Meta-analysis of the literature or of individual patient data: is there a difference? *Lancet* 1993;341:418-22.
39. Burke DL, Ensor J, Riley RD. Meta-analysis using individual participant data: one-stage and two-stage approaches, and why they may differ. *Statist Med* 2016;36:855-75. doi: 10.1002/sim.7141
40. Debray TP, Moons KG, Abo-Zaid GM, et al. Individual participant data meta-analysis for a binary outcome: one-stage or two-stage? *PloS One* 2013; 8.
41. Munn Z, Stern C, Aromataris E, et al. What kind of systematic review should I conduct? A proposed typology and guidance for systematic reviewers in the medical and health sciences. *BMC Med Res Methodol* 2018; 18.
42. Stewart LA, Clarke M, Rovers M, et al. Preferred reporting items for a systematic review and meta-analysis of individual participant data: the PRISMA-IPD statement. *JAMA* 2015;313(16):1657-65. doi: 10.1001/jama.2015.3656
43. Stroup DF, Berlin JA, Morton SC, et al. Meta-analysis of observational studies in epidemiology. *JAMA* 2000;283:2008-12.
44. Higgins JPT, Altman DG, Sterne JAC. Chapter 8: Assessing risk of bias in included studies In: Higgins JPT, Altman DG, Sterne JAC, eds. Cochrane Handbook for Systematic Reviews of Interventions version 520. www.training.cochrane.org/handbook: The Cochrane Collaboration, 2017.
45. Liberati A, Altman DG, Tetzlaff J, et al. The PRISMA statement for reporting systematic reviews and meta-analyses of studies that evaluate health care interventions: explanation and elaboration. *PLOS Med* 2009; 6(7).
46. Deeks JJ, Higgins JPT, Altman DG. Chapter 9: Analysing data and undertaking meta-analyses. In: Higgins JPT, Churchill R, Chandler J, et al., eds. Cochrane Handbook for Systematic Reviews of Interventions version 520. www.training.cochrane.org/handbook: The Cochrane Collaboration, 2017.
47. Hukkelhoven CWPM, Steyerberg EW, Rampen AJJ, et al. Patient age and outcome following severe traumatic brain injury: an analysis of 5600 patients. *J Neurosurg* 2003;99:666-73.
48. Riley RD, Simmonds MC, Look MP. Evidence synthesis combining individual patient data and aggregate data: a systematic review identified current practice and possible methods. *J Clin Epidemiol* 2007;60:431-39. doi: 10.1016/j.jclinepi.2006.09.009
49. Steyerberg EW, Kievit J, de Mol Van Otterloo JC. Perioperative mortality of elective abdominal aortic aneurysm surgery. A clinical prediction rule based on literature and individual patient data. *Arch Intern Med* 1995;155:1998-2004.
50. Ly LP, Sartorius G, Hull L, et al. Accuracy of calculated free testosterone formulae in men. *Clin Endocrinol* 2010;73:382-88. doi: 10.1111/j.1365-2265.2010.03804.x
51. Borenstein M, Hedges LV, Higgins JPT, et al. Introduction to Meta-Analysis. West Sussex: John Wiley & Sons Ltd 2009.
52. Fisher R. Statistical methods for research workers (2nd ed.). New York: Hafner 1928.
53. Pearson K. On a correction needful in the case of the correlation ratio. *Biometrika* 1911;8:254-56.
54. Keiding N, Anderson PK, Klein JP. The role of frailty models and accelerated failure time models in describing heterogeneity due to omitted covariates. *Statist Med* 1997;16:215-24.
55. Vaupel JW, Manton KG, Stallard E. The impact of heterogeneity in individual frailty on the dynamics of mortality. *Demography* 1979;16:439-54.
56. Yashin A, Vaupel J, Iachine I. Correlated individual frailty: An advantageous approach to survival analysis of bivariate data. *Math Popul Stud* 1995;5:145-59.

For peer review only - http://bmjopen.bmj.com/site/about/guidelines.xhtml

57. Ahmed I, Sutton AJ, Riley RD. Assessment of publication bias, selection bias, and unavailable data in meta-analyses using individual participant data: a database survey. *BMJ* 2012; 344.
58. Shamseer L, Moher D, Clarke M, et al. Preferred reporting items for systematic review and meta-analysis protocols (PRISMA-P) 2015: elaboration and explanation. *BMJ* 2015; 350.
59. Sterne JAC, Sutton AJ, Ioannidis JPA, et al. Recommendations for examining an interpretting funnel plot asymmetry in meta-analyses of randomised controlled trials. *BMJ* 2011; 342.
60. Fried LP, Borhani NO, Enright P, et al. The Cardiovascular Health Study: Design and Rationale. *Ann Epidemiol* 1991;1:263-76.
61. Grant JF, Martin SA, Taylor AW, et al. Cohort Profile: The Men Androgen Inflammation Lifestyle Environment and Stress (MAILES) Study. *Int J Epidemiol* 2014;43:1040-53. doi: 10.1093/ije/dyt064
62. Kannel WB, Feinleib M, McNamara PM, et al. An investigation of coronary heart disease in families: The Framingham Offspring Study. *Am J Epidemiol* 1979;110:281-90.
63. Knuiman MW, Jamrozik K, Welborn TA, et al. Age and secular trends in risk factors for cardiovascular disease in Busselton. *Aust J Public Health* 1995;19:375-82.
64. Lee DM, O'Neill TW, Pye SR, et al. The European Male Ageing Study (EMAS): design, methods and recruitment. *Int J Androl* 2008;32:11-24. doi: 10.1111/j.1365-2605.2008.00879.x
65. Norman PE, Flicker L, Almeida OP, et al. Cohort Profile: The Health In Men Study (HIMS). *Int J Epidemiol* 2009;38:48-52. doi: 10.1093/ije/dyn041
66. Orwoll E, Blank JB, Barrett-Connor E, et al. Design and baseline characteristics of the osteoporotic fractures in men (MrOS) study — A large observational study of the determinants of fracture in older men. *Contemp Clin Trials* 2005;26:569-85. doi: 10.1016/j.cct.2005.05.006
67. The ARIC Investigators. The Atherosclerosis Risk In Communities (ARIC) study: design and objectives. *Am J Epidemiol* 1989;129:687-702.
68. Araujo AB, Dixon JM, Suarez EA, et al. Endogenous Testosterone and Mortality in Men: A Systematic Review and Meta-Analysis. *J Clin Endocrinol Metab* 2011;96:3007-19. doi: 10.1210/jc.2011-1137
69. Atlantis E, Fahey P, Cochrane B, et al. Endogenous testosterone level and testosterone supplementation therapy in chronic obstructive pulmonary disease (COPD): a systematic review and meta-analysis. *BMJ Open* 2013; 3.
70. Boyle P, Koechlin A, Bota M, et al. Endogenous and exogenous testosterone and the risk of prostate cancer and increased prostatespecific antigen (PSA) level: a meta-analysis. *BJU Int* 2016;118:731-41. doi: 10.1111/bju.13417
71. Brand JS, Rovers MM, Yeap BB, et al. Testosterone, Sex Hormone-Binding Globulin and the Metabolic Syndrome in Men: An Individual Participant Data Meta-Analysis of Observational Studies. *PloS One* 2014; 9.
72. Brand JS, van der Tweel I, Grobbee DE, et al. Testosterone, sex hormone-binding globulin and the metabolic syndrome: a systematic review and meta-analysis of observational studies. *Int J Epidemiol* 2010;40:189-207. doi: 10.1093/ije/dyq158
73. Claps M, Petrelli F, Caffo O, et al. Testosterone Levels and Prostate Cancer Prognosis: Systematic Review and Meta-analysis. *Clin Genitourin Cancer* 2018;16:165-75. doi: 10.1016/j.clgc.2018.01.005
74. Corona G, Monami M, Rastrelli G, et al. Testosterone and Metabolic Syndrome: A Meta-Analysis Study. *J Sex Med* 2011;8:272-83. doi: 10.1111/j.1743-6109.2010.01991.x
75. Haddad RM, Kennedy CC, Caples SM, et al. Testosterone and cardiovascular risk in men: A systematic review and meta-analysis of randomised placebo-controlled trials. *Mayo Clin Proc* 2007;82:29-39.
76. Holmegard HN, Nordestgaard BG, Jensen GB, et al. Sex hormones and ischemic stroke: a prospective cohort study and meta-analyses. *J Clin Endocrinol Metab* 2016;101:69-78. doi: 10.1210/jc.2015-2687

For peer review only - http://bmjopen.bmj.com/site/about/guidelines.xhtml

77. Isidori AM, Giannetta E, Gianfrilli D, et al. Effects of testosterone on sexual function in men: results of a meta-analysis. *Clin Endocrinol* 2005;63:381-94. doi: 10.1111/j.1365-2265.2005.02350.x
78. Lv W, Du N, Liu Y, et al. Low Testosterone Level and Risk of Alzheimer's Disease in the Elderly Men: a Systematic Review and Meta-Analysis. *Mol Neurobiol* 2016;53:2679-84. doi: 10.1007/s12035-015-9315-y
79. Ruige JB, Mahmoud AM, Bacquer DD, et al. Endogenous testosterone and cardiovascular disease in healthy men: a meta-analysis. *Heart* 2010;97:870-75. doi: 10.1136/hrt.2010.210757
80. Gordis L. Epidemiology. 4th ed. Philadelphia: Saunders 2009.

For peer review only

For peer review only - http://bmjopen.bmj.com/site/about/guidelines.xhtml

TABLES

Table 1. Variables planned to be included in IPD meta-analysis modelling.¶

Type	Analysis 1	Analysis 2	Analysis 3	Analysis 4
**Outcomes / DV**	Androgen concentration§	Incident CVD*	Incident deaths (cancer*)	Incident dementia
		Incident deaths (CVD*)	Incident cancer* (diagnoses)	Baseline cognition
		Incident deaths (all-cause)		Change in cognition
**Focal predictor**	-	Androgen concentration§	Androgen concentration§	Androgen concentration§
**Covariates / IV**				
**Demographic**	age	age	age	age
	education level	education level	education level	education level
	ethnicity	ethnicity	ethnicity	ethnicity
	marital status	marital status	marital status	marital status
	site	site	site	site
**Risk factors and comorbidities**	alcohol consumption	alcohol consumption	alcohol consumption	alcohol consumption
	BMI, waist	BMI, waist	BMI, waist	BMI, waist
	physical activity	physical activity	physical activity	physical activity
	smoking status	smoking status	smoking status	smoking status
	BP, hypertension	BP, hypertension	BP, hypertension	BP, hypertension
	general health	general health	general health	general health
		atrial fibrillation		
	prevalent CVD	prevalent CVD		
	prevalent cancer*		prevalent cancer*	
	prevalent dementia			prevalent dementia
	baseline cognition			
	COPD	COPD		
	diabetes	diabetes	diabetes	diabetes
	cholesterol, LDL, HDL	cholesterol, LDL, HDL		
	creatinine level	creatinine level		
	lipid lowering medications	lipid lowering medications		
	anxiety			anxiety
	depression			depression
	psychotropic drug use			psychotropic drug use

Abbreviations: BMI = body mass index; BP= blood pressure; CVD = Cardiovascular Disease; DV = dependent variable; COPD = Chronic Obstructive Pulmonary Disease; IV = independent variables.

¶ = Black font: highly desirable; Green font: only if available.

For peer review only - http://bmjopen.bmj.com/site/about/guidelines.xhtml

§ = Androgens: total testosterone, dihydrotestosterone, estradiol, luteinising hormone, sex hormone binding globulin.

* = Subgroup analyses are also planned. For CVD outcomes: Heart Failure, Myocardial Infarction, Stroke. For cancer outcomes: colorectal cancer, lung cancer, prostate cancer.

For peer review only

For peer review only - http://bmjopen.bmj.com/site/about/guidelines.xhtml

Supplementary Table S1. Example search strategy for systematic review: AIMS study.

The following is an example of a preliminary search conducted in MEDLINE.

1. Testosterone/ or Androgens/
2. (testosterone or androgen* or sex hormone* or sex steroid*).ti.
3. (testosterone or androgen*).ab.
4. cardiovascular diseases/ or heart diseases/ or heart failure/ or vascular diseases/ or stroke/ or myocardial infarction/ or coronary disease/ or cerebrovascular disorders/
5. (cardiovascular or stroke or myocardial infarction or heart failure).ti.
6. neoplasms/ or colorectal neoplasms/ or lung neoplasms/ or prostatic neoplasms/
7. cancer.ti.
8. mortality/ or mortality.ti.
9. dementia/ or cognition/ or dementia.ti. or cognit*.ti.
10. Aging/psychology or Neuropsychological Tests/
11. 1 or 2 or 3
12. 4 or 5 or 6 or 7 or 8 or 9 or 10
13. 11 and 12
14. longitudinal studies/ or prospective studies/ or follow-up studies/ or cohort studies/
15. (prospective or follow-up or cohort study or longitudinal study).ti,ab.
16. 14 or 15
17. 13 and 16
18. (exogenous or replacement or therapy or hormone treatment).ti.
19. Hormone Replacement Therapy/
20. 18 or 19
21. 17 not 20
22. limit 21 to humans
23. limit 22 to english language
24. limit 23 to (adaptive clinical trial or address or autobiography or bibliography or biography or case reports or clinical trial, all or clinical trial, phase i or clinical trial, phase ii or clinical trial, phase iii or clinical trial, phase iv or clinical trial, veterinary or clinical trials, veterinary as topic or clinical trial protocol or clinical trial or comment or controlled clinical trial or dictionary or editorial or lecture or legislation or meta analysis or practice guideline or pragmatic clinical trial or published erratum or randomized controlled trial or retracted publication or "retraction of publication" or "review" or "scientific integrity review" or "systematic review")
25. Retrospective Studies/ or Case-Control Studies/ or (retrospective analysis or case-control).ti.
26. 24 or 25
27. 23 not 26

Notes:

Terms with a trailing "/" are MeSH terms and those with a trailing "*" are truncated search strings. This search strategy has been submitted for registration on PROSPERO [Systematic Review Protocol ID 139668, submitted on 23 July 2019 and is presently being assessed]. Beforehand, a search of PROSPERO was conducted for another suitable strategy but none

For peer review only

were found. However, the above strategy is based upon one that has been used for a similar study.[1]

References cited

1. Holmegard HN, Nordestgaard BG, Jensen GB, Tybjaerg-Hansen A, Benn M. Sex hormones and ischemic stroke: a prospective cohort study and meta-analyses. J Clin Endocrinol Metab. 2016;101:69-78.

For peer review only

PRISMA-IPD Checklist of items to include when reporting a systematic review and meta-analysis of individual participant data (IPD): the Androgens In Men Study (AIMS).

PRISMA-IPD Section/topic	Item No	Checklist item	Reported on page
**Title**			
Title	1	Identify the report as a systematic review and meta-analysis of individual participant data.	1
**Abstract**			
Structured summary	2	Provide a structured summary including as applicable:	4-5
		**Background**: state research question and main objectives, with information on participants, interventions, comparators and outcomes.	
		**Methods**: report eligibility criteria; data sources including dates of last bibliographic search or elicitation, noting that IPD were sought; methods of assessing risk of bias.	
		**Results**: provide number and type of studies and participants identified and number (%) obtained; summary effect estimates for main outcomes (benefits and harms) with confidence intervals and measures of statistical heterogeneity. Describe the direction and size of summary effects in terms meaningful to those who would put findings into practice.	
		**Discussion:** state main strengths and limitations of the evidence, general interpretation of the results and any important implications.	
		**Other:** report primary funding source, registration number and registry name for the systematic review and IPD meta-analysis.	
**Introduction**			
Rationale	3	Describe the rationale for the review in the context of what is already known.	6-7
Objectives	4	Provide an explicit statement of the questions being addressed with reference, as applicable, to participants, interventions, comparisons, outcomes and study design (PICOS). Include any hypotheses that relate to particular types of participant-level subgroups.	8
**Methods**			
Protocol and registration	5	Indicate if a protocol exists and where it can be accessed. If available, provide registration information including registration number and registry name. Provide publication details, if applicable.	5,9
Eligibility criteria	6	Specify inclusion and exclusion criteria including those relating to participants, interventions, comparisons, outcomes, study design and characteristics (e.g. years when conducted, required minimum follow-up). Note whether these were applied at the study or individual level i.e. whether eligible participants were included (and ineligible participants excluded) from a study that included a wider population than specified by the review inclusion criteria. The rationale for criteria should be stated.	8-9

For peer review only - http://bmjopen.bmj.com/site/about/guidelines.xhtml

PRISMA-IPD Section/topic	Item No	Checklist item	Reported on page
**Title**			
Identifying studies - information sources	7	Describe all methods of identifying published and unpublished studies including, as applicable: which bibliographic databases were searched with dates of coverage; details of any hand searching including of conference proceedings; use of study registers and agency or company databases; contact with the original research team and experts in the field; open adverts and surveys. Give the date of last search or elicitation.	9
Identifying studies - search	8	Present the full electronic search strategy for at least one database, including any limits used, such that it could be repeated.	9, Suppl. Data
Study selection processes	9	State the process for determining which studies were eligible for inclusion.	9
Data collection processes	10	Describe how IPD were requested, collected and managed, including any processes for querying and confirming data with investigators. If IPD were not sought from any eligible study, the reason for this should be stated (for each such study).	10-11
		If applicable, describe how any studies for which IPD were not available were dealt with. This should include whether, how and what aggregate data were sought or extracted from study reports and publications (such as extracting data independently in duplicate) and any processes for obtaining and confirming these data with investigators.	
Data items	11	Describe how the information and variables to be collected were chosen. List and define all study level and participant level data that were sought, including baseline and follow-up information. If applicable, describe methods of standardising or translating variables within the IPD datasets to ensure common scales or measurements across studies.	10-11
IPD integrity	A1	Describe what aspects of IPD were subject to data checking (such as sequence generation, data consistency and completeness, baseline imbalance) and how this was done.	10-11
Risk of bias assessment in individual studies.	12	Describe methods used to assess risk of bias in the individual studies and whether this was applied separately for each outcome. If applicable, describe how findings of IPD checking were used to inform the assessment. Report if and how risk of bias assessment was used in any data synthesis.	10-11
Specification of outcomes and effect measures	13	State all treatment comparisons of interests. State all outcomes addressed and define them in detail. State whether they were pre-specified for the review and, if applicable, whether they were primary/main or secondary/additional outcomes. Give the principal measures of effect (such as risk ratio, hazard ratio, difference in means) used for each outcome.	12-16
Synthesis methods	14	Describe the meta-analysis methods used to synthesise IPD. Specify any statistical methods and models used. Issues should include (but are not restricted to):<br>• Use of a one-stage or two-stage approach.<br>• How effect estimates were generated separately within each study and combined across studies (where applicable).	13-16

For peer review only - http://bmjopen.bmj.com/site/about/guidelines.xhtml

PRISMA-IPD Section/topic	Item No	Checklist item	Reported on page
**Title**			
		• Specification of one-stage models (where applicable) including how clustering of patients within studies was accounted for.<br>• Use of fixed or random effects models and any other model assumptions, such as proportional hazards.<br>• How (summary) survival curves were generated (where applicable).<br>• Methods for quantifying statistical heterogeneity (such as I^2 and τ^2).<br>• How studies providing IPD and not providing IPD were analysed together (where applicable).<br>• How missing data within the IPD were dealt with (where applicable).	
Exploration of variation in effects	A2	If applicable, describe any methods used to explore variation in effects by study or participant level characteristics (such as estimation of interactions between effect and covariates). State all participant-level characteristics that were analysed as potential effect modifiers, and whether these were pre-specified.	13-16
Risk of bias across studies	15	Specify any assessment of risk of bias relating to the accumulated body of evidence, including any pertaining to not obtaining IPD for particular studies, outcomes or other variables.	10, 16
Additional analyses	16	Describe methods of any additional analyses, including sensitivity analyses. State which of these were pre-specified.	8, 16
**Results**			
Study selection and IPD obtained	17	Give numbers of studies screened, assessed for eligibility, and included in the systematic review with reasons for exclusions at each stage. Indicate the number of studies and participants for which IPD were sought and for which IPD were obtained. For those studies where IPD were not available, give the numbers of studies and participants for which aggregate data were available. Report reasons for non-availability of IPD. Include a flow diagram.	TBA
Study characteristics	18	For each study, present information on key study and participant characteristics (such as description of interventions, numbers of participants, demographic data, unavailability of outcomes, funding source, and if applicable duration of follow-up). Provide (main) citations for each study. Where applicable, also report similar study characteristics for any studies not providing IPD.	TBA
IPD integrity	A3	Report any important issues identified in checking IPD or state that there were none.	TBA
Risk of bias within studies	19	Present data on risk of bias assessments. If applicable, describe whether data checking led to the up-weighting or down-weighting of these assessments. Consider how any potential bias impacts on the robustness of meta-analysis conclusions.	TBA
Results of individual studies	20	For each comparison and for each main outcome (benefit or harm), for each individual study report the number of eligible participants for which data were obtained and show simple summary data for each intervention group (including, where applicable, the number of events), effect estimates and confidence intervals. These may be tabulated or included on a forest	TBA

For peer review only - http://bmjopen.bmj.com/site/about/guidelines.xhtml

PRISMA-IPD Section/topic	Item No	Checklist item	Reported on page
**Title**			
		plot.	
Results of syntheses	21	Present summary effects for each meta-analysis undertaken, including confidence intervals and measures of statistical heterogeneity. State whether the analysis was pre-specified, and report the numbers of studies and participants and, where applicable, the number of events on which it is based.	TBA
		When exploring variation in effects due to patient or study characteristics, present summary interaction estimates for each characteristic examined, including confidence intervals and measures of statistical heterogeneity. State whether the analysis was pre-specified. State whether any interaction is consistent across trials.	
		Provide a description of the direction and size of effect in terms meaningful to those who would put findings into practice.	
Risk of bias across studies	22	Present results of any assessment of risk of bias relating to the accumulated body of evidence, including any pertaining to the availability and representativeness of available studies, outcomes or other variables.	TBA
Additional analyses	23	Give results of any additional analyses (e.g. sensitivity analyses). If applicable, this should also include any analyses that incorporate aggregate data for studies that do not have IPD. If applicable, summarise the main meta-analysis results following the inclusion or exclusion of studies for which IPD were not available.	TBA
**Discussion**			
Summary of evidence	24	Summarise the main findings, including the strength of evidence for each main outcome.	TBA
Strengths and limitations	25	Discuss any important strengths and limitations of the evidence including the benefits of access to IPD and any limitations arising from IPD that were not available.	TBA
Conclusions	26	Provide a general interpretation of the findings in the context of other evidence.	TBA
Implications	A4	Consider relevance to key groups (such as policy makers, service providers and service users). Consider implications for future research.	TBA
**Funding**			
Funding	27	Describe sources of funding and other support (such as supply of IPD), and the role in the systematic review of those providing such support.	20-21

For peer review only

A1 – A3 denote new items that are additional to standard PRISMA items. A4 has been created as a result of re-arranging content of the standard PRISMA statement to suit the way that systematic review IPD meta-analyses are reported.

© Reproduced with permission of the PRISMA IPD Group, which encourages sharing and reuse for non-commercial purposes

For peer review only

For peer review only - http://bmjopen.bmj.com/site/about/guidelines.xhtml

For peer review only

For peer review only - http://bmjopen.bmj.com/site/about/guidelines.xhtml

BMJ Open

Androgens In Men Study (AIMS): protocol for meta-analyses of individual participant data investigating associations of androgens with health outcomes in men.

Journal:	*BMJ Open*
Manuscript ID	bmjopen-2019-034777.R1
Article Type:	Protocol
Date Submitted by the Author:	28-Nov-2019

Complete List of Authors:	Yeap, Bu; University of Western Australia, Medical School; Fiona Stanley Hospital, Department of Endocrinology and Diabetes<br>Marriott, Ross; University of Western Australia, School of Population and Global Health<br>Adams, Robert; Flinders University, Adelaide Institute for Sleep Health<br>Antonio, Leen; KU Leuven, Clinical and Experimental Endocrinology<br>Ballantyne, Christie; Baylor College of Medicine, Internal Medicine<br>Bhasin, Shalender; Harvard Medical School<br>Cawthon, PM; California Pacific Medical Center Research Institute, San Francisco Coordinating Center<br>Couper, David; University of North Carolina at Chapel Hill, Gillings School of Global Public Health<br>Dobs, Adrian; Johns Hopkins University, School of Medicine, Division of Endocrinology, Diabetes and Metabolism<br>Flicker, Leon; University of Western Australia, WA Centre for Health & Ageing<br>Karlsson, Magnus; Lund University, Department of Clinical Sciences and Orthopedic Surgery<br>Martin, Sean A.; The University of Adelaide, Freemasons Foundation Centre for Men's Health<br>Matsumoto, AM; VA Puget Sound Health Care System, Geriatric Research, Education and Clinical Center; University of Washington System, Medicine<br>Mellström, Dan ; University of Gothenburg, Centre for Bone and Arthritis Research at the Sahlgrenska Academy, Institute of Medicine<br>Norman, Paul; University of Western Australia, Medical School<br>Ohlsson , C; University of Gothenburg, Centre for Bone and Arthritis Research at the Sahlgrenska Academy, Institute of Medicine<br>Orwoll, Eric; Oregon Health & Science University<br>O'Neill, Terence; The University of Manchester & NIHR Manchester Biomedical Research Centre, Centre for Epidemiology Versus Arthritis, Faculty of Biology, Medicine and Health; Manchester Academic Health Science Centre, Manchester University NHS Foundation Trust<br>Shores, Molly; VA Puget Sound Health Care System; University of Washington, School of Medicine, Department of Psychiatry and Behavioral Sciences<br>Travison, Thomas; Beth Israel Deaconess Medical Center, Institute for Aging Research, Hebrew SeniorLife; Harvard Medical School<br>Vanderschueren, Dirk; Katholieke Universiteit Leuven, Department of

	Chronic Diseases, Metabolism and Ageing (CHROMETA), Laboratory of Clinical and Experimental Endocrinology<br>Wittert, Gary; The University of Adelaide, Freemasons Centre for Men's Health<br>Wu, Frederick; The University of Manchester, Division of Diabetes, Endocrinology and Gastroenterology<br>Murray, Kevin; University of Western Australia, School of Population and Global Health
**Primary Subject Heading**:	Diabetes and endocrinology
Secondary Subject Heading:	Epidemiology
Keywords:	Sex steroids & HRT < DIABETES & ENDOCRINOLOGY, EPIDEMIOLOGY, General endocrinology < DIABETES & ENDOCRINOLOGY

I, the Submitting Author has the right to grant and does grant on behalf of all authors of the Work (as defined in the below author licence), an exclusive licence and/or a non-exclusive licence for contributions from authors who are: i) UK Crown employees; ii) where BMJ has agreed a CC-BY licence shall apply, and/or iii) in accordance with the terms applicable for US Federal Government officers or employees acting as part of their official duties; on a worldwide, perpetual, irrevocable, royalty-free basis to BMJ Publishing Group Ltd ("BMJ") its licensees and where the relevant Journal is co-owned by BMJ to the co-owners of the Journal, to publish the Work in this journal and any other BMJ products and to exploit all rights, as set out in our licence.

The Submitting Author accepts and understands that any supply made under these terms is made by BMJ to the Submitting Author unless you are acting as an employee on behalf of your employer or a postgraduate student of an affiliated institution which is paying any applicable article publishing charge ("APC") for Open Access articles. Where the Submitting Author wishes to make the Work available on an Open Access basis (and intends to pay the relevant APC), the terms of reuse of such Open Access shall be governed by a Creative Commons licence – details of these licences and which Creative Commons licence will apply to this Work are set out in our licence referred to above.

Other than as permitted in any relevant BMJ Author's Self Archiving Policies, I confirm this Work has not been accepted for publication elsewhere, is not being considered for publication elsewhere and does not duplicate material already published. I confirm all authors consent to publication of this Work and authorise the granting of this licence.

For peer review only

Androgens In Men Study (AIMS): protocol for meta-analyses of individual participant data investigating associations of androgens with health outcomes in men.

Corresponding author

Dr Bu B. Yeap MBBS, FRACP, PhD, Harry Perkins Institute of Medical Research, 11 Robin Warren Drive, Murdoch 6150, Western Australia, Australia.

Email: bu.yeap@uwa.edu.au; Telephone: +61 8 6151 1149

Authors:

Bu B. Yeap, Medical School, The University of Western Australia, Perth 6009, Australia.

Ross J. Marriott, School of Population and Global Health, The University of Western Australia, Perth 6009, Australia.

Robert J. Adams, Adelaide Institute for Sleep Health, Flinders University, Bedford Park 5042, Australia.

Leen Antonio, Clinical and Experimental Endocrinology, KU Leuven, Leuven, Belgium.

Christie M. Ballantyne, Internal Medicine, Baylor College of Medicine, Houston, Texas, USA.

Shalender Bhasin, Harvard Medical School, Boston, Massachusetts, USA.

Peggy M. Cawthon, San Francisco Coordinating Center, California Pacific Medical Center Research Institute, San Francisco, California, USA.

David J. Couper, Gillings School of Global Public Health, University of North Carolina at Chapel Hill, Chapel Hill, North Carolina, USA.

Adrian S. Dobs, School of Medicine, Division of Endocrinology, Diabetes and Metabolism, Johns Hopkins University, Baltimore, Maryland, USA.

For peer review only

For peer review only - http://bmjopen.bmj.com/site/about/guidelines.xhtml

Leon Flicker, WA Centre for Health & Ageing, The University of Western Australia, Perth 6009, Australia.

Magnus Karlsson, Department of Clinical Sciences and Orthopedic Surgery, Lund University, Lund, Sweden.

Sean A. Martin, Freemasons Foundation Centre for Men's Health, The University of Adelaide, Adelaide 5000, Australia.

Alvin M. Matsumoto, Geriatric Research, Education and Clinical Center, VA Puget Sound Health Care System, Seattle, Washington, USA.

Dan Mellström, Centre for Bone and Arthritis Research at the Sahlgrenska Academy, Institute of Medicine, University of Gothenburg, Goteburg, Sweden.

Paul E. Norman, Medical School, The University of Western Australia, Fremantle 6160, Australia.

Claes Ohlsson, Centre for Bone and Arthritis Research at the Sahlgrenska Academy, Institute of Medicine, University of Gothenburg, Goteburg, Sweden.

Eric S. Orwoll, Oregon Health & Science University, Portland, Oregon, USA.

Terence W. O'Neill, Centre for Epidemiology Versus Arthritis, Faculty of Biology, Medicine and Health, The University of Manchester & NIHR Biomedical Research Centre, Manchester, United Kingdom.

Molly M. Shores, VA Puget Sound Health Care System, Seattle, Washington, United States.

Thomas G. Travison, Institute for Aging Research, Hebrew SeniorLife, Beth Israel Deaconess Medical Center, Boston, Massachusetts, USA.

Dirk Vanderschueren, Department of Chronic Diseases, Metabolism and Ageing (CHROMETA), Laboratory of Clinical and Experimental Endocrinology, Katholieke Universiteit Leuven, Leuven, Flanders, Belgium.

For peer review only

For peer review only - http://bmjopen.bmj.com/site/about/guidelines.xhtml

Gary A. Wittert, Freemasons Foundation Centre for Men's Health, The University of Adelaide, Adelaide 5000, Australia.

Frederick C. W. Wu., Division of Diabetes, Endocrinology and Gastroenterology, The University of Manchester, Manchester, United Kingdom.

Kevin Murray, School of Population and Global Health, The University of Western Australia, Perth 6009, Australia.

<u>Word count, excluding title page, abstract, references, acknowledgements, contributions, figures and tables:</u>

3,436 words.

For peer review only

For peer review only - http://bmjopen.bmj.com/site/about/guidelines.xhtml

ABSTRACT

Introduction

This study aims to clarify the role(s) of endogenous sex hormones to influence health outcomes in men, specifically to define the associations of plasma testosterone with incidence of cardiovascular events, cancer, dementia and mortality risk, and to identify factors predicting testosterone concentrations. Data will be accrued from at least three Australian, two European, and four North American population-based cohorts involving approximately 20,000 men.

Methods and analysis

Eligible studies include prospective cohort studies with baseline testosterone concentrations measured using mass spectrometry and five years of follow-up data on incident cardiovascular events, mortality, cancer diagnoses or deaths, new onset dementia, or decline in cognitive function recorded. Data for men, who were not taking androgens or drugs suppressing testosterone production, metabolism, or action; and had no prior orchidectomy, are eligible. No date range was set for searches. Aggregate level data will be sought where individual participant data (IPD) is not available. One-stage IPD random-effects meta-analyses will be performed, using linear mixed models, generalised linear mixed models and either stratified or frailty-augmented Cox regression models. Heterogeneity in estimates from different studies will be quantified and bias investigated using funnel plots. Effect size estimates will be presented in forest plots and non-negligible heterogeneity and bias investigated using subgroup or meta-regression analyses.

Ethics and dissemination

Ethics approvals obtained for each of the participating cohorts state that participants have consented to have their data collected and used for research purposes. There are four planned

For peer review only - http://bmjopen.bmj.com/site/about/guidelines.xhtml

research articles, with each involving a separate set of IPD meta-analyses (articles will investigate different, distinct outcomes).

Registration PROSPERO Registration No. CRD42019139668.

STRENGTHS AND LIMITATIONS OF THIS STUDY

- The IPD meta-analyses are likely to have higher statistical power than and provide greater scope to control for important confounders and risk factors than previous meta-analyses on this topic.
- Investigators from nine large prospective cohort studies (each with N > 1,000 participants) have agreed to collaborate., with additional studies to be identified from systematic review.
- Harmonization will be required for some variables (e.g., physical activity, alcohol consumption) that are recorded differently among the component studies, and aggregate-level data will be sought where IPD-level data are not available.
- As this is an observational study, it will not fully eliminate the possibility of confounding influences of unadjusted effects.
- However, unlike a randomised controlled trial, this study will provide a more comprehensive characterisation of temporal relationships between baseline androgen concentrations and health outcomes in community-dwelling adult males.

For peer review only - http://bmjopen.bmj.com/site/about/guidelines.xhtml

INTRODUCTION

As men grow older, testosterone production and circulating concentrations of testosterone decline while comorbidities accumulate.[1-4] Older men, even those in very good health, have lower circulating testosterone concentrations compared with healthy young men.[5, 6] Although results have been inconsistent, an increasing number of studies have reported associations of low endogenous testosterone concentration with poorer health outcomes, especially in older men. For instance, studies that have used liquid chromatography tandem mass spectrometry, widely regarded as the reference method for the measurement of total testosterone concentrations,[7] have reported associations of lower endogenous testosterone concentrations with: (i) cardiovascular disease and all-cause mortality in some cases[8-15] but not others[8, 16-20]; (ii) some cancers but not others[21, 22]; and (iii) dementia[23, 24] but not laboratory measures of cognitive function.[25] Therefore it remains unclear whether testosterone is a biomarker of ill-health or a causal factor for diseases of ageing.

Currently, testosterone treatment is recommended for men who have symptoms and signs of androgen deficiency and low testosterone concentrations, due to disease of the hypothalamus, pituitary or testes (organic or pathological hypogonadism).[26-29] Randomised controlled trials of testosterone treatment in men aged 65 and older with low-normal testosterone concentrations without organic hypogonadism have shown modest benefits on sexual function, anaemia, self-reported physical function and mobility, and volumetric bone density, but not on some objective measures of cognition over 12 to 36 months.[30-34] The effect of testosterone on major adverse cardiovascular events remains unclear.[35, 36] However, the selection criteria of these trials was such that the screening to enrolment ratio was 65:1, a highly selected population of older men.[32, 33, 36] Importantly, the trials were neither large enough nor long enough to determine the effects of testosterone on major adverse

For peer review only

cardiovascular events, development of dementia, bone fractures, and mortality.[36] Therefore, a meta-analysis of data from prospective cohort studies, with extended follow-up periods, provides opportunities for better understanding of the temporal profiles of the postulated associations between endogenous testosterone concentration and incident health outcomes. Meta-analyses of Individual Participant Data (IPD) are generally preferable to meta-analyses of aggregate data in that they typically have higher statistical power and provide scope to control for important confounders and risk factors.[37, 38] Furthermore, one-stage IPD meta-analyses are preferable to two-stage approaches because the former uses an exact likelihood to directly model the distribution of IPD, offers the convenience of using a standard set of diagnostic tools to assess model fit, and can arguably provide greater flexibility, in terms of options for statistical modelling.[39, 40]

The Androgens In Men Study (AIMS), an international collaboration of prospective cohort studies, will examine the associations of sex hormones (comprising testosterone and dihydrotestosterone as the major androgens, and estradiol as the major estrogen, in the circulation) with health outcomes that are major sources of morbidity and mortality in middle-aged and older men. The group will perform a series of IPD meta-analyses to clarify the influence of sex hormone exposures on major health outcomes, including heart attack, stroke and cardiovascular deaths, cancer, new-onset dementia, and all-cause mortality, as well as provide information on social, demographic and behavioural factors that are associated with endogenous testosterone concentrations. This work will characterise robustly the associations of several sex hormones with health outcomes in men in general over and above the study-specific estimates, and thus clarify the role of androgens as biomarkers for, or causal contributors to, men's health. The work outlined in this document will be conducted from 1 February 2019 to 30 November 2020.

For peer review only - http://bmjopen.bmj.com/site/about/guidelines.xhtml

Objectives

The Androgens In Men Study (AIMS) will establish an international collaboration of existing cohort studies to clarify the relation of endogenous sex hormones with major health outcomes in men. PEO (Population, Exposure, Outcomes) characteristics include: adult men in the general community; an exposure of endogenous circulating sex hormone concentration, primarily testosterone, as the principal male sex hormone or androgen; and a prospective cohort study type, with incident health outcomes including incidence of cardiovascular disease events, mortality, cancers, and dementia. The specific objectives of the AIMS study are: to investigate associations between variables representing social, demographic, and behavioural factors with the measured concentration of testosterone in the blood of men (Analysis 1); to examine the associations between testosterone concentrations and subsequent incidence of cardiovascular events, cardiovascular deaths, and all-cause mortality in men (Analysis 2); to examine the associations between testosterone concentrations and subsequent mortality from (and, if available, diagnoses of) common cancers in men (Analysis 3); to examine the associations between testosterone concentrations and cognitive impairment and incident dementia in men (Analysis 4). Analysis 2 will evaluate myocardial infarction, stroke and heart failure, deaths due to cardiovascular disease, and the composite endpoint of major adverse cardiovascular events (MACE) comprising non-fatal myocardial infarction, non-fatal stroke, and deaths due to cardiovascular disease. Analysis 3 will evaluate outcomes of deaths due to and diagnoses of colorectal cancer, lung cancer and prostate cancer.

For peer review only - http://bmjopen.bmj.com/site/about/guidelines.xhtml

METHODS AND ANALYSIS

IPD meta-analyses will be conducted to understand the associations between testosterone and a range of associated major health outcomes in men. IPD meta-analyses have been selected as the most suitable approach because: (i) the required aggregate data (AD) are not available from each of the cohorts in published literature; (ii) IPD meta-analyses typically have higher statistical power than AD meta-analyses and provide scope for controlling for important confounders and risk factors.[37, 38] Where possible, methods will adhere to the guidelines for Preferred Reporting Items for Systematic Reviews and Meta-Analyses of IPD data (PRISMA-IPD; completed checklist is provided in Supplementary Material) and the Meta-analysis Of Observational Studies in Epidemiology (MOOSE).[41-43]

For peer review only

Studies selected for inclusion in IPD meta-analyses

Studies will be identified from a systematic review using online search tools for mainstream published (MEDLINE, EMBASE) and grey literature (OpenGrey, Mednar) studies. Eligible studies include prospective cohort studies with plasma or serum testosterone concentrations measured using mass spectrometry with at least five years of follow-up data, with incident cardiovascular, cancer, mortality, dementia, or cognitive events recorded (see Supplementary Table S1 for an example of search criteria to be used). The search strategy selects for articles based on words or Medical Subject Headings (MeSH) terms matching the relevant exposure (example steps 1-3), outcomes (example steps 4-10), and study type (example steps 14-15), then, depending on the search tool, filters down to more relevant studies, with exclusions of clinical trials and of studies on non-humans (example steps 18-27), with no date range restrictions. Ahead of the systematic review, nine eligible studies (cohorts) had expressed interest to collaborate: three from Australia (Busselton Health Study BHS, Health In Men Study HIMS, Men Androgen Inflammation Lifestyle Environment and Stress study

For peer review only - http://bmjopen.bmj.com/site/about/guidelines.xhtml

MAILES); two from Europe (European Male Ageing Study EMAS, Osteoporotic Fractures in Men Study MrOS - Sweden); and four from the USA (Atherosclerosis Risk in Communities study ARIC, Cardiovascular Health Study CHS, The Framingham Heart Study FHS, Osteoporotic Fractures in Men Study MrOS - USA). Investigators from eight of these studies have confirmed availability of suitable IPD-level data, provisional on approvals from their respective Publication and Steering Committees. If it is not possible to obtain IPD-level data from selected studies, we will request suitable AD-level data.

Data provision, merging, harmonisation, and storage

The project manager will liaise directly with the nominated contact person for each cohort study to identify, specifically, which variables and set of observations will be suitable to request. A data request will then be submitted to the data custodian for each study. Requested variables will be labelled as either "highly desirable" or "only if available", in order to prioritise efforts in obtaining the key variables for analyses and to acknowledge the differing availability of variables among studies. A list of variable names, definitions, and attributes, including numbers of rows and columns in each data file(s) will also be requested to be provided separately. Methods for ascertaining outcome and comorbidity status will be requested, which can be used to indicate the relative quality of diagnostic information (e.g. ICD-coded diagnoses from hospital inpatient admissions versus self-report information). File transfer will be achieved via encrypted file transfer (or other sufficiently secure method).

Once each dataset has been received by the project manager, the original file(s) will be saved and date-stamped in a secure central repository. All subsequent manipulations will be completed using copies of the original file, with syntax saved as script files. Variable definitions will be checked, variables inspected for missing values, and variable properties

For peer review only - http://bmjopen.bmj.com/site/about/guidelines.xhtml

and value ranges assessed to identify possible outliers. A table of summary statistics will be calculated and, where possible, analyses run and compared with published values to check for data consistency. The nature of any discrepancies identified from these checks will need to be understood, and possibly resolved, prior to proceeding with the meta-analyses.[42, 44, 45]

Depending on the extent of missing-ness, missing data will be suitably imputed.[45, 46] For each analysis, we will conduct multiple imputations using a method that approximates as close as possible to the substantive model.[47] The method will likely vary depending on the analysis[48] and therefore we will consider adapting either a Fully Conditional Specification (e.g., [49]) or Joint Modelling framework (e.g., [50]) to each case, as is appropriate. The quality of imputations will be quantified by using re-imputed values to calculate the posterior predictive p-values of relevant quantities.[51] Results from each of the multiply-imputed datasets will be suitably pooled to obtain final estimates, standard errors, and 95% confidence intervals.[52]

Prior to the merging of datasets, a variable to identify each source study will be appended as the new first column. Variable formats will be checked and corrected for consistency and anonymised participant identifier codes for uniqueness across all studies. Harmonization will be required for some variables (e.g., physical activity, alcohol consumption) that are recorded differently by the different component studies. Where possible, AD datasets will be used to reconstruct IPD-level data (that is, partially-reconstructed IPD) prior to merging.[53-55] Should this not be possible, the IPD-level data will be aggregated and summary estimates made with and without AD-level data as sensitivity analyses. It is also possible that some studies might have outcomes available for some analyses, but not others; in these cases, we

For peer review only

For peer review only - http://bmjopen.bmj.com/site/about/guidelines.xhtml

252 will preferentially use IPD-level data when available but also seek to use AD-level data from those other studies when available. There is no requirement to use IPD-level data from the same studies across all analyses.

256 All IPD-level data will be accessible to only the approved staff from a secure on-site facility, from rooms that are kept locked when unattended, and with remote access not permitted. IPD-level data are not to be printed in hard copy and will only be presented at aggregate level. Data analysed for this study will be retained for five years after the last of all proposed analyses are published and will then be destroyed. A post-analysis retention period is required to enable publication and possible scrutiny of findings. Disposal will be carried out according to best practice.

Data items

The full list of generic variables to include in meta-analyses are presented in Table 1. Analysis 1 will model relationships of total testosterone concentrations, as measured in serum or plasma samples (dependent variable), with key demographic variables and risk factors for disease (predictor variables). Time-to-event variables obtained from follow-up data will be analysed in Analysis 2 and 3 (outcomes), and their associations with testosterone concentrations (focal predictor) and other potential confounders and risk factors (predictor variables). Records of dementia diagnoses (physician or otherwise categorised) and relative cognitive function (for example, from test scores) will be analysed in Analysis 4, and their associations with testosterone concentrations and other potential confounders and risk factors. Analyses of relationships with other sex hormones instead of testosterone, including dihydrotestosterone, estradiol, luteinising hormone (LH), and Sex Hormone Binding Globulin

For peer review only - http://bmjopen.bmj.com/site/about/guidelines.xhtml

(SHBG), will be conducted where sufficient data is available. For exploratory analyses, free testosterone, the amount in the circulation which is not protein-bound, will be calculated from measured total testosterone and SHBG.[56] Covariates were selected to include those used in previous studies, as well as those that are typically recorded. The full list of proposed variables to include in the meta-analyses are presented in Table 1.

Statistical analysis

Since the datasets to be analysed are sampled from different populations, a random-effects meta-analysis is appropriate, as it acknowledges that effects will vary among studies due to differences in local factors.[57] One-stage IPD meta-analyses will be performed. Each IPD meta-analysis will involve fitting a model with study estimated as either a fixed or random term, to account for related observations within studies, and testosterone modelled as random slopes (when modelled as a continuous predictor) or intercepts (when modelled as a categorical predictor), to harmonised, merged data from all cohorts. The underlying statistical model and estimates of effect size will be specific to each of the proposed analyses and are outlined as follows.

Analysis 1: Factors associated with testosterone concentrations in men and characterisation of reference ranges.

Linear Mixed Models (LMMs) or Generalised Linear Mixed Models (GLMMs) will be used to model the relation between the predictors (independent variables) and each hormonal variable (testosterone, dihydrotestosterone, estradiol, LH, SHBG) as five separate IPD meta-analyses. Suspected non-linear relationships, at the scale of the linear predictor, will be investigated and modelled appropriately (e.g., splines with pre-set knot locations and linear

For peer review only - http://bmjopen.bmj.com/site/about/guidelines.xhtml

boundary constraints). Measures of effect size may include (but are not limited to): η^2,[58, 59] Pearson's r, standardised mean difference to the reference level (categorical predictors), standardised difference for an increase in one standard deviation (continuous predictors). In the case of non-linear relations, we will graphically describe the relationship with comparisons made with appropriate reference points. Reference ranges will be derived based on the distributions of testosterone in healthy men.

Analysis 2: Associations between testosterone concentrations and subsequent incidence of cardiovascular events, cardiovascular deaths, and all-cause mortality.

Cox proportional hazards models will be used to assess the effect of testosterone level on the incident risk of each outcome, with separate IPD meta-analyses conducted for each outcome (myocardial infarction, stroke, heart failure, deaths due to cardiovascular disease, and MACE) and each hormonal variable (testosterone, dihydrotestosterone, estradiol, LH, SHBG). Component study will be modelled either as a stratified variable[39] or as a random term, and testosterone as a random term, using frailty models, which are a class of survival models that incorporate random effects.[60-62] Participants with prevalent cardiovascular disease at baseline will be excluded. The length of follow-up will also be standardised among studies in order to maximise data from all datasets, whilst minimising the prospect for variable lengths-to-follow-up among studies introducing additional heterogeneity into results.[46]

Multivariable versions of each of these models will also be fitted, with additional predictors for potential confounders and risk factors included (Table 1). Non-linear associations for continuous variables will be modelled using natural splines with pre-specified knots and

For peer review only - http://bmjopen.bmj.com/site/about/guidelines.xhtml

324 linear boundary constraints. The (standardised) measure of effect size used will be the hazard
325 ratio. Subgroup analyses will be conducted separately for each of three specific types of
326 cardiovascular disease (outcomes): myocardial infarction, stroke and heart failure.

327

328 *Analysis 3: Association between testosterone level and subsequent incidence of cancer.*

329 Cox proportional hazards models will be used to assess the effect of testosterone
330 concentrations on the incident risk of cancer deaths and, if available, of cancer diagnoses.
331 IPD meta-analyses separately conducted for each of these outcomes and each hormonal
332 variable as focal predictor (testosterone, dihydrotestoterone, estradiol, LH, SHBG).
333 Component study will be modelled either as a stratified variable[39] or as a random term, and
334 testosterone as a random term, using frailty models.[60-62] Participants with prevalent cancer
335 diagnosis at baseline will be excluded. The length of follow-up will be standardised among
336 studies in order to maximise data from all datasets, whilst minimising the prospect for
337 variable lengths-to-follow-up among studies introducing additional heterogeneity into
338 results.[46]

339

340 Multivariable versions of each of these models will also be fitted, with additional predictors
341 for potential confounders and risk factors included (Table 1). Non-linear associations for
342 continuous variables will be modelled using natural splines with pre-specified knots and
343 linear boundary constraints. The (standardised) measure of effect size used will be the hazard
344 ratio. Subgroup analyses will be conducted separately for each of three common types of
345 cancers in men (outcomes): colorectal cancer, lung cancer, prostate cancer. For these
346 analyses, men with the relevant cancer type at baseline will be excluded from that specific
347 analysis. Thus men with prevalent colorectal cancer (but not other cancer types) will be

For peer review only

excluded in the analysis of incident colorectal cancer; similarly for the analyses of incident lung and prostate cancer, men with lung or prostate cancer at baseline will be excluded.

Analysis 4: Associations of testosterone levels with cognitive impairment and incident dementia in men.

LMMs and GLMMs will be used to model the association of testosterone concentrations with cognitive impairment (cross-sectional analyses of baseline data). Cox proportional hazards models will be used to assess the effect of testosterone concentrations on the incident risk of dementia. Men with prevalent dementia will be excluded from this analysis. We will also ask for follow-up cognition test scores, and if available, will run an analysis of changes in cognition test scores from baseline as an outcome. Separate IPD meta-analyses will be conducted for each hormonal variable as a focal predictor (testosterone, dihydrotestosterone, estradiol, LH, SHBG). Component study will be modelled as a fixed or random intercept term in LMMs and GLMMs and as a stratified factor or frailty model random term in Cox regressions. Multivariable versions of each of these models will also be fitted, with additional predictors for potential confounders and risk factors included (Table 1). Suspected non-linear relationships, at the scale of the linear predictor, will be investigated and modelled using splines with pre-specified knots and boundary constraints. Measures of effect size may include: η^2, Pearson's r, standardised mean difference to the reference level (categorical predictors), standardised difference for an increase in one standard deviation (continuous predictors), odds ratio for LMMs and GLMMs and hazard ratio for Cox regressions.

Throughout all analyses, contour-enhanced funnel plots will be constructed to visually assess patterns in estimates of effect sizes and precision among studies, to investigate heterogeneity and possible meta-biases.[63-65] The relative amount of heterogeneity will be estimated

For peer review only

(e.g., using I^2) and forest plots presented. Subgroup or meta-regression analyses may be conducted if pronounced heterogeneity is estimated.[57]

Patient and public involvement

This IPD meta-analysis will use existing secondary data. Patients and public were not involved in the design, recruitment or conduct of this IPD meta-analysis. The results of this study will be shared with the primary investigators of the shared studies and disseminated as publications in open-access journals.

ETHICS AND DISSEMINATION

Ethics approvals obtained for each of the participating cohorts state that participants have consented to have their data collected and used for research purposes. Furthermore, there are no expected harms or risks to participants, as data have already been collected within each individual cohort, under existing ethics approvals. De-identified data will be collated and analysed, with no new procedures planned for participants. The AIMS study has been assessed as exempt from ethics review by the Human Research Ethics Office at the University of Western Australia (file reference number RA/4/20/5014).

DISCUSSION

Although several published meta-analyses have investigated associations of endogenous testosterone with health outcomes in men,[66-77] none have conducted IPD meta-analyses of health outcomes as planned for this study. A previous IPD meta-analysis focussed on the outcome of metabolic syndrome.[69] Results from this study will improve upon previously published estimates from individual studies, in terms of the generalisability of findings. Estimates from the IPD meta-analyses are also likely to be more reliable than those published

For peer review only - http://bmjopen.bmj.com/site/about/guidelines.xhtml

from conventional meta-analyses because they typically have higher statistical power and provide scope for controlling for important confounders and risk factors.[37, 38] Uncertainty will undoubtedly remain due to the possibility of confounding influences of unadjusted effects. However, unlike a randomised controlled trial, this study avoids the need to subject individuals to interventions, and provides more comprehensive characterisation of temporal relationships between baseline testosterone concentrations and a range of key health outcomes.[78]

Accordingly, it is hoped that the AIMS collaboration will ultimately complement the research efforts and outputs from multiple prospective cohort studies by drawing upon the collective body of evidence to clarify the role of endogenous sex hormone levels on major health outcomes in men. It is possible that this work might also elucidate new understanding, arising from improved scope for fitting more complex models due to increased statistical power, or from patterns detected in subgroup or meta-regression analyses. Clinically, research outputs will be used to identify the scope and optimal recruitment criteria for future trials of testosterone therapy. These data will also allow reference ranges for testosterone in men across ages and geographical locations to be refined, to inform recommendations for clinical practice more generally.

Acknowledgements

We thank Östen Ljunggren of the Department of Medical Sciences, Uppsala University and Liesbeth Vandenput of the Centre for Bone and Arthritis Research at the Sahlgrenska Academy Institute of Medicine, University of Gothenburg, Mary Lou Biggs, Terena Solomons, Jos Tournoy, Mark Divitni, Matthew Knuiman, John Acres, the staff and participants of the ARIC study, the Busselton Population Medical Research Foundation for

For peer review only

For peer review only - http://bmjopen.bmj.com/site/about/guidelines.xhtml

access to the data, the Cardiovascular Health Study, the EMAS study, the Health In Men Study, the MAILES study, the MrOS Study, for their important contributions.

Collaborators

The Androgens In Men Study: (1) Busselton Health Study: Busselton Population Medical Research Institute (Inc.), Department of Pulmonary Physiology and Sleep Medicine, Sir Charles Gairdner Hospital, Nedlands WA 6009, Australia (2) Health In Men Study (HIMS): Western Australian Centre for Health and Ageing, University of Western Australia, Level 6 Medical Research Foundation Building, Royal Perth Hospital, Rear 50 Murray Street, Perth, Western Australia 6000, Australia. (3) Men Androgen Inflammation Lifestyle Environment and Stress (MAILES) study: Freemasons Foundation Centre for Men's Health, University of Adelaide, 254 North Tce, Adelaide, Australia. (4) European Male Ageing Study (EMAS): The University of Manchester, Stopford Building, Oxford Road, Manchester, M13 9PT, UK. (5) Osteoporotic Fractures in Men (MrOS) Study Sweden: Department of Clinical Sciences and Orthopedic Surgery, Lund University, Skåne University Hospital, Malmö, Sweden; MrOS Study USA: California Pacific Medical Center Research Institute, San Francisco Coordinating Center, 550 16th street, San Francisco, CA 94143. (6) Atherosclerosis Risk in Communities study (ARIC): School of Public Health, U. of North Carolina, Suite 2013, NCNB Plaza, 137 E. Franklin St, Chapel Hill, NC 27514. (7) Cardiovascular Health Study: National Heart, Lung, and Blood Institute, Bethesda, MD. (8) The Framingham Heart Study, Boston University School of Medicine, 72 E. Concord Street, B-601, Boston, MA 02118.

For peer review only

For peer review only - http://bmjopen.bmj.com/site/about/guidelines.xhtml

Authors' contributions

BBY, KM, LA, SB, ASD, AMM, CO, EO, TWO, DV, GAW, contributed to the study concept and design. BBY, KM, RJM prepared the initial draft of the manuscript, including literature review, list of variables and statistical methods. BBY, RJM, RJA, LA, CMB, SB, PMC, DJC, ASD, LF, MK, SAM, AMM, DM, PEN, CO, ESO, TWO, MMS, TGT, DV, GAW, FCWW, KM were involved in subsequent revisions to the protocol and manuscript, and approved this submission.

Funding

AIMS is funded by a Western Australian Health Translation Network (WAHTN) Medical Research Future Fund Rapid Applied Translation Grant (2018). The ARIC study is funded by has been funded in whole or in part with Federal funds from the National Heart, Lung, and Blood Institute, National Institutes of Health, Department of Health and Human Services, under Contract nos. (HHSN268201700001I, HHSN268201700002I, HHSN268201700003I, HHSN268201700005I, HHSN268201700004I) and Dr. Ballantyne received NIH grant support through R01HL134320 The 1994/1995 Busselton Health Survey was supported by the Western Australian Health Promotion Foundation. The Cardiovascular Health Study was supported by contracts HHSN268201200036C, HHSN268200800007C, HHSN268201800001C, N01HC55222, N01HC85079, N01HC85080, N01HC85081, N01HC85082, N01HC85083, N01HC85086, and grants U01HL080295 and U01HL130114 from the National Heart, Lung, and Blood Institute (NHLBI), with additional contribution from the National Institute of Neurological Disorders and Stroke (NINDS). Additional support was provided by R01AG023629 from the National Institute on Aging (NIA). A full list of principal CHS investigators and institutions can be found at CHS-NHLBI.org. The EMAS study is funded by The Commission of the European Communities Fifth Framework

For peer review only - http://bmjopen.bmj.com/site/about/guidelines.xhtml

Programme "Quality of Life and Management of Living Resources" Grant No. QLK6-CT-2001-00 258 and supported by Arthritis Research UK. The Framingham Heart Study of the National Heart Lung and Blood Institute of the National Institutes of Health and Boston University School of Medicine has been funded in whole or in part with Federal funds from the National Heart, Lung, and Blood Institute, National Institutes of Health, Department of Health and Human Services, under Contract No. 75N92019D00031. The HIMS study is funded by project grants from the National Health and Medical Research Council of Australia. The MAILES study is funded by a project grant from the National Health and Medical Research Council of Australia (627227). The MrOS Sweden study is funded by The Swedish Research Council and Swedish ALF funding. The Osteoporotic Fractures in Men (MrOS) Study is supported by National Institutes of Health funding. The following institutes provide support: the National Institute on Aging (NIA), the National Institute of Arthritis and Musculoskeletal and Skin Diseases (NIAMS), the National Center for Advancing Translational Sciences (NCATS), and NIH Roadmap for Medical Research under the following grant numbers: U01 AG027810, U01 AG042124, U01 AG042139, U01 AG042140, U01 AG042143, U01 AG042145, U01 AG042168, U01 AR066160, and UL1 TR000128.

For peer review only

Disclaimer

The funders played no role in the study design, data collection, data analysis or interpretation, writing of the manuscript or the decision to submit the article for publication. All authors are accountable for all aspects of the work in ensuring that questions related to the accuracy or integrity of any part of the work are appropriately investigated and resolved. The content is solely the responsibility of the authors and does not necessarily represent the official views of the National Institutes of Health.

For peer review only - http://bmjopen.bmj.com/site/about/guidelines.xhtml

Competing interests

None declared.

Patient consent

Each of the component studies have addressed participant/patient consent permissions; please refer to respective component studies for details.[79-86]

Ethics approval

The AIMS study has been assessed as exempt from ethics review by the Human Ethics office at the University of Western Australia (file reference number RA/4/20/5014). Each of the component studies had obtained ethics approvals; please refer to respective component studies for details.[79-86]

Provenance and peer review

Not commissioned; externally peer reviewed.

For peer review only

For peer review only - http://bmjopen.bmj.com/site/about/guidelines.xhtml

References cited

1. Handelsman DJ, Yeap BB, Flicker L, et al. Age-specific population centiles for androgen status in men. *Eur J Endocrinol* 2015;173(6):809-17. doi: 10.1530/EJE-15-0380
2. Hsu B, Cumming RG, Hirani V, et al. Temporal trend in androgen status and androgen-sensitive outcomes in older men. *J Clin Endocrinol Metab* 2016;101(4):1836-46. doi: 10.1210/jc.2015-3810
3. Shi Z, Araujo AB, Martin S, et al. Longitudinal changes in testosterone over five years in community-dwelling men. *J Clin Endocrinol Metab* 2013;98(8):3289-97. doi: 10.1210/jc.2012-3842
4. Yeap BB, Manning L, Chubb SAP, et al. Progressive impairment of testicular endocrine function in ageing men: testosterone and dihydrotestosterone decrease, and luteinizing hormone increases, in men transitioning from the 8th to 9th decades of life. *Clin Endocrinol* 2018;88:88-95. doi: 10.1111/cen.13484
5. Sikaris K, McLachlan RI, Kazlauskas R, et al. Reproductive hormone reference intervals for healthy fertile young men: evaluation of automated platform assays. *J Clin Endocrinol Metab* 2005;90(11):5928-36. doi: 10.1210/jc.2005-0962
6. Yeap BB, Alfonso H, Chubb SAP, et al. Reference ranges and determinants of testosterone, dihydrotestosterone, and estradiol levels measured using liquid chromatography-tandem mass spectrometry in a population-based cohort of older men. *J Clin Endocrinol Metab* 2012;97(11):4030-39. doi: 10.1210/jc.2012-2265
7. Handelsman DJ, Wartofsky L. Requirement for Mass Spectrometry Sex Steroid Assays in the Journal of Clinical Endocrinology and Metabolism. *J Clin Endocrinol Metab* 2013;98:3971-73. doi: 10.1210/jc.2013-3375
8. Hsu B, Cumming RG, Naganathan V, et al. Temporal Changes in Androgens and Estrogens Are Associated With All-Cause and Cause-Specific Mortality in Older Men. *J Clin Endocrinol Metab* 2016;101:2201-10. doi: 10.1210/jc.2016-1025
9. Ohlsson C, Barrett-Connor E, Bhasin S, et al. High Serum Testosterone Is Associated With Reduced Risk of Cardiovascular Events in Elderly Men. *J Am Coll Cardiol* 2011;58:1674-81. doi: 10.1016/j.jacc.2011.07.019
10. Pye SR, Huhtaniemi IT, Finn JD, et al. Late-Onset Hypogonadism and Mortality in Aging Men. *J Clin Endocrinol Metab* 2014;99:1357-66. doi: 10.1210/jc.2013-2052
11. Tivesten A, Mellstrom D, Jutberger H, et al. Low serum testosterone and high serum estradiol associate with lower extremity peripheral arterial disease in elderly men. *J Am Coll Cardiol* 2007;50:1070-76. doi: 10.1016/j.jacc.2007.04.088
12. Tivesten A, Vandenput L, Labrie F, et al. Low serum testosterone and estradiol predict mortality in elderly men. *J Clin Endocrinol Metab* 2009;94:2482-88. doi: 10.1210/jc.2008-2650
13. Yeap BB, Alfonso H, Chubb SAP, et al. In older men, higher plasma testosterone or dihydrotestosterone is an independent predictor for reduced incidence of stroke but not myocardial infarction. *J Clin Endocrinol Metab* 2014;99(12):4565-73. doi: 10.1210/jc.2014-2664
14. Yeap BB, Hyde Z, Almeida OP, et al. Lower testosterone levels predict incident stroke and transient ischemic attack in older men. *J Clin Endocrinol Metab* 2009;94(7):2353-59. doi: 10.1210/jc.2008-2416
15. Yeap BB, Knuiman MW, Divitni ML, et al. Differential associations of testosterone, dihydrotestosterone and oestradiol with physical, metabolic and health-related factors in community-dwelling men aged 17–97 years from the Busselton Health Survey. *Clin Endocrinol* 2014;81:100-08.
16. Chan YX, Knuiman MW, Hung J, et al. Neutral associations of testosterone, dihydrotestosterone and estradiol with fatal and non-fatal cardiovascular events, and mortality in men aged 17–97 years. *Clin Endocrinol* 2016;85:575-82. doi: 10.1111/cen.13089

For peer review only - http://bmjopen.bmj.com/site/about/guidelines.xhtml

561 17. Shores MM, Arnold AM, Biggs ML, et al. Testosterone and dihydrotestosterone and incident ischaemic stroke in men in the Cardiovascular Health Study. *Clin Endocrinol* 2014;81:746-53. doi: 10.1111/cen.12452
564 18. Shores MM, Biggs ML, Arnold AM, et al. Testosterone, Dihydrotestosterone, and Incident Cardiovascular Disease and Mortality in the Cardiovascular Health Study. *J Clin Endocrinol Metab* 2014;99:2061-68. doi: 10.1210/jc.2013-3576
567 19. Srinath R, Golden SH, Carson KA, et al. Endogenous Testosterone and its Relationship to Preclinical and Clinical Measures of Cardiovascular Disease in the Atherosclerosis Risk in Communities Study. *J Clin Endocrinol Metab* 2015;100:1602-08. doi: 10.1210/jc.2014-3934
570 20. Srinath R, Gottesman RF, Golden SH, et al. Association Between Endogenous Testosterone and Cerebrovascular Disease in the ARIC Study (Atherosclerosis Risk in Communities). *Stroke* 2016;47:2682-88. doi: 10.1161/STROKEAHA.116.014088
573 21. Chan YX, Knuiman MW, Divitni ML, et al. Lower circulating androgens are associated with overall cancer risk and prostate cancer risk in men aged 25-84 years from the Busselton Health Study. *Horm Cancer* 2018;9:391-98. doi: 10.1007/s12672-018-0346-5
576 22. Daniels NA, Nielson CM, Hoffman AR, et al. Sex hormones and the risk of incident prostate cancer. *Urology* 2010;76:1034-14. doi: 10.1016/j.urology.2010.01.086
578 23. Ford AH, Yeap BB, Flicker L, et al. Sex hormones and incident dementia in older men: The health in men study. *Psychoneuroendocrinol* 2018;98:139-47. doi: 10.1016/j.psyneuen.2018.08.013
580 24. Ford AH, Yeap BB, Flicker L, et al. Prospective longitudinal study of testosterone and incident depression in older men: The Health In Men Study. *Psychoneuroendocrinology* 2016;64:57-65. doi: 10.1016/j.psyneuen.2015.11.012
583 25. LeBlanc ES, Wang PY, Janowsky JS, et al. Association between sex steroids and cognition in elderly men. *Clin Endocrinol* 2010;72:393-403. doi: 10.1111/j.1365-2265.2009.03692.x
585 26. Yeap BB, Wu FCW. Clinical practice update on testosterone therapy for male hypogonadism: contrasting perspectives to optimize care. *Clin Endocrinol* 2019;90:56-65. doi: 10.1111/cen.13888
587 27. Yeap BB, Grossmann M, McLachlan RI, et al. Endocrine Society of Australia position statement on male hypogonadism (part 2): treatment and therapeutic considerations. *Med J Aust* 2016;205:228-31. doi: 10.5694/mja16.00448
590 28. Yeap BB, Grossmann M, McLachlan RI, et al. Endocrine Society of Australia position statement on male hypogonadsm (part 1): assessment and indications for testosterone therapy. *Med J Aust* 2016;205:173-78. doi: 10.5694/mja16.00393
593 29. Bhasin S, Brito JP, Cunningham GR, et al. Testosterone therapy in men with hypogonadism: an Endocrine Society* Clinical Practice Guideline. *J Clin Endocrinol Metab* 2018;103(5):1715-44. doi: 10.1210/jc.2018-00229
596 30. Resnick SM, Matsumoto AM, Stephens-Shields AJ, et al. Testosterone treatment and cognitive function in older men with low testosterone and age-associated memory impairment. *JAMA* 2017;317:717-27. doi: 10.1001/jama.2016.21044
599 31. Roy CN, Snyder PJ, Stephens-Shields AJ, et al. Association of testosterone levels with anemia in older men: A controlled clinical trial. *JAMA Intern Med* 2017;177:480-90. doi: 10.1001/jamainternmed.2016.9540
602 32. Snyder PJ, Bhasin S, Cunningham GR, et al. Effects of testosterone treatment in older men. *N Engl J Med* 2016;374:611-24. doi: 10.1056/NEJMoa1506119
604 33. Snyder PJ, Bhasin S, Cunningham GR, et al. Lessons from the testosterone trials. *Endocr Rev* 2018;39:369-86. doi: 10.1210/er.2017-00234
606 34. Snyder PJ, Kopperdahl DL, Stephens-Shields AJ, et al. Effect of testosterone treatment on volumetric bone density and strength in older men with low testosterone: A controlled clinical trial. *JAMA Intern Med* 2017;177:471-79. doi: 10.1001/jamainternmed.2016.9539
609 35. Budoff M, Ellenberg SS, Lewis CE, et al. Testosterone treatment and coronary artery plaque volume in older men with low testosterone. *JAMA* 2017;317:708-16. doi: 10.1001/jama.2016.21043

For peer review only - http://bmjopen.bmj.com/site/about/guidelines.xhtml

36. Yeap BB, Page ST, Grossmann M. Testosterone treatment in older men: clinical implications and unresolved questions from the Testosterone Trials. *Lancet Diabetes Endocrinol* 2018;6:659-72. doi: 10.1016/S2213-8587(17)30416-3

37. Riley RD, Lambert PC, Abo-Zaid G. Meta-analysis of individual participant data: rationale, conduct, and reporting. *BMJ* 2010; 340.

38. Stewart LA, Parmar MKB. Meta-analysis of the literature or of individual patient data: is there a difference? *Lancet* 1993;341:418-22.

39. Burke DL, Ensor J, Riley RD. Meta-analysis using individual participant data: one-stage and two-stage approaches, and why they may differ. *Statist Med* 2016;36:855-75. doi: 10.1002/sim.7141

40. Debray TP, Moons KG, Abo-Zaid GM, et al. Individual participant data meta-analysis for a binary outcome: one-stage or two-stage? *PloS One* 2013; 8.

41. Munn Z, Stern C, Aromataris E, et al. What kind of systematic review should I conduct? A proposed typology and guidance for systematic reviewers in the medical and health sciences. *BMC Med Res Methodol* 2018; 18.

42. Stewart LA, Clarke M, Rovers M, et al. Preferred reporting items for a systematic review and meta-analysis of individual participant data: the PRISMA-IPD statement. *JAMA* 2015;313(16):1657-65. doi: 10.1001/jama.2015.3656

43. Stroup DF, Berlin JA, Morton SC, et al. Meta-analysis of observational studies in epidemiology. *JAMA* 2000;283:2008-12.

44. Higgins JPT, Altman DG, Sterne JAC. Chapter 8: Assessing risk of bias in included studies In: Higgins JPT, Altman DG, Sterne JAC, eds. Cochrane Handbook for Systematic Reviews of Interventions version 520. www.training.cochrane.org/handbook: The Cochrane Collaboration, 2017.

45. Liberati A, Altman DG, Tetzlaff J, et al. The PRISMA statement for reporting systematic reviews and meta-analyses of studies that evaluate health care interventions: explanation and elaboration. *PLOS Med* 2009; 6(7).

46. Deeks JJ, Higgins JPT, Altman DG. Chapter 9: Analysing data and undertaking meta-analyses. In: Higgins JPT, Churchill R, Chandler J, et al., eds. Cochrane Handbook for Systematic Reviews of Interventions version 520. www.training.cochrane.org/handbook: The Cochrane Collaboration, 2017.

47. van Buuren S. Flexible Imputation of Missing Data, Second Edition. Milton: CRC Press LLC 2018.

48. Graham J. Missing data analysis: making it work in the real world. *Annu Rev Psychol* 2009;60:549-76. doi: 10.1146/annurev.psych.58.110405.085530

49. van Buuren S, Brand J, Groothuis-Oushoorn K, et al. Fully conditional specification in multivariate imputation. *J Stat Comput Simul* 2006;76:1049-64. doi: 10.1080/10629360600810434

50. Kenward M, Carpenter J. Multiple imputation of quantitative data. Multiple Imputation and Its Application. Chicester: Wiley 2013:77-89.

51. He Y, Zaslavsky A. Diagnosing imputation models by applying target analyses to posterior replicates of completed data. *Statist Med* 2010;31:1-18. doi: 10.1002/sim.4413

52. Rubin DB. Multiple imputation for nonresponse in surveys. New York: Wiley 1987.

53. Hukkelhoven CWPM, Steyerberg EW, Rampen AJJ, et al. Patient age and outcome following severe traumatic brain injury: an analysis of 5600 patients. *J Neurosurg* 2003;99:666-73.

54. Riley RD, Simmonds MC, Look MP. Evidence synthesis combining individual patient data and aggregate data: a systematic review identified current practice and possible methods. *J Clin Epidemiol* 2007;60:431-39. doi: 10.1016/j.jclinepi.2006.09.009

55. Steyerberg EW, Kievit J, de Mol Van Otterloo JC. Perioperative mortality of elective abdominal aortic aneurysm surgery. A clinical prediction rule based on literature and individual patient data. *Arch Intern Med* 1995;155:1998-2004.

56. Ly LP, Sartorius G, Hull L, et al. Accuracy of calculated free testosterone formulae in men. *Clin Endocrinol* 2010;73:382-88. doi: 10.1111/j.1365-2265.2010.03804.x

For peer review only

For peer review only - http://bmjopen.bmj.com/site/about/guidelines.xhtml

57. Borenstein M, Hedges LV, Higgins JPT, et al. Introduction to Meta-Analysis. West Sussex: John Wiley & Sons Ltd 2009.
58. Fisher R. Statistical methods for research workers (2nd ed.). New York: Hafner 1928.
59. Pearson K. On a correction needful in the case of the correlation ratio. *Biometrika* 1911;8:254-56.
60. Keiding N, Anderson PK, Klein JP. The role of frailty models and accelerated failure time models in describing heterogeneity due to omitted covariates. *Statist Med* 1997;16:215-24.
61. Vaupel JW, Manton KG, Stallard E. The impact of heterogeneity in individual frailty on the dynamics of mortality. *Demography* 1979;16:439-54.
62. Yashin A, Vaupel J, Iachine I. Correlated individual frailty: An advantageous approach to survival analysis of bivariate data. *Math Popul Stud* 1995;5:145-59.
63. Ahmed I, Sutton AJ, Riley RD. Assessment of publication bias, selection bias, and unavailable data in meta-analyses using individual participant data: a database survey. *BMJ* 2012; 344.
64. Shamseer L, Moher D, Clarke M, et al. Preferred reporting items for systematic review and meta-analysis protocols (PRISMA-P) 2015: elaboration and explanation. *BMJ* 2015; 350.
65. Sterne JAC, Sutton AJ, Ioannidis JPA, et al. Recommendations for examining an interpretting funnel plot asymmetry in meta-analyses of randomised controlled trials. *BMJ* 2011; 342.
66. Araujo AB, Dixon JM, Suarez EA, et al. Endogenous Testosterone and Mortality in Men: A Systematic Review and Meta-Analysis. *J Clin Endocrinol Metab* 2011;96:3007-19. doi: 10.1210/jc.2011-1137
67. Atlantis E, Fahey P, Cochrane B, et al. Endogenous testosterone level and testosterone supplementation therapy in chronic obstructive pulmonary disease (COPD): a systematic review and meta-analysis. *BMJ Open* 2013; 3.
68. Boyle P, Koechlin A, Bota M, et al. Endogenous and exogenous testosterone and the risk of prostate cancer and increased prostatespecific antigen (PSA) level: a meta-analysis. *BJU Int* 2016;118:731-41. doi: 10.1111/bju.13417
69. Brand JS, Rovers MM, Yeap BB, et al. Testosterone, Sex Hormone-Binding Globulin and the Metabolic Syndrome in Men: An Individual Participant Data Meta-Analysis of Observational Studies. *PloS One* 2014; 9.
70. Brand JS, van der Tweel I, Grobbee DE, et al. Testosterone, sex hormone-binding globulin and the metabolic syndrome: a systematic review and meta-analysis of observational studies. *Int J Epidemiol* 2010;40:189-207. doi: 10.1093/ije/dyq158
71. Claps M, Petrelli F, Caffo O, et al. Testosterone Levels and Prostate Cancer Prognosis: Systematic Review and Meta-analysis. *Clin Genitourin Cancer* 2018;16:165-75. doi: 10.1016/j.clgc.2018.01.005
72. Corona G, Monami M, Rastrelli G, et al. Testosterone and Metabolic Syndrome: A Meta-Analysis Study. *J Sex Med* 2011;8:272-83. doi: 10.1111/j.1743-6109.2010.01991.x
73. Haddad RM, Kennedy CC, Caples SM, et al. Testosterone and cardiovascular risk in men: A systematic review and meta-analysis of randomised placebo-controlled trials. *Mayo Clin Proc* 2007;82:29-39.
74. Holmegard HN, Nordestgaard BG, Jensen GB, et al. Sex hormones and ischemic stroke: a prospective cohort study and meta-analyses. *J Clin Endocrinol Metab* 2016;101:69-78. doi: 10.1210/jc.2015-2687
75. Isidori AM, Giannetta E, Gianfrilli D, et al. Effects of testosterone on sexual function in men: results of a meta-analysis. *Clin Endocrinol* 2005;63:381-94. doi: 10.1111/j.1365-2265.2005.02350.x
76. Lv W, Du N, Liu Y, et al. Low Testosterone Level and Risk of Alzheimer's Disease in the Elderly Men: a Systematic Review and Meta-Analysis. *Mol Neurobiol* 2016;53:2679-84. doi: 10.1007/s12035-015-9315-y
77. Ruige JB, Mahmoud AM, Bacquer DD, et al. Endogenous testosterone and cardiovascular disease in healthy men: a meta-analysis. *Heart* 2010;97:870-75. doi: 10.1136/hrt.2010.210757
78. Gordis L. Epidemiology. 4th ed. Philadelphia: Saunders 2009.

For peer review only - http://bmjopen.bmj.com/site/about/guidelines.xhtml

79. Fried LP, Borhani NO, Enright P, et al. The Cardiovascular Health Study: Design and Rationale. *Ann Epidemiol* 1991;1:263-76.
80. Grant JF, Martin SA, Taylor AW, et al. Cohort Profile: The Men Androgen Inflammation Lifestyle Environment and Stress (MAILES) Study. *Int J Epidemiol* 2014;43:1040-53. doi: 10.1093/ije/dyt064
81. Kannel WB, Feinleib M, McNamara PM, et al. An investigation of coronary heart disease in families: The Framingham Offspring Study. *Am J Epidemiol* 1979;110:281-90.
82. Knuiman MW, Jamrozik K, Welborn TA, et al. Age and secular trends in risk factors for cardiovascular disease in Busselton. *Aust J Public Health* 1995;19:375-82.
83. Lee DM, O'Neill TW, Pye SR, et al. The European Male Ageing Study (EMAS): design, methods and recruitment. *Int J Androl* 2008;32:11-24. doi: 10.1111/j.1365-2605.2008.00879.x
84. Norman PE, Flicker L, Almeida OP, et al. Cohort Profile: The Health In Men Study (HIMS). *Int J Epidemiol* 2009;38:48-52. doi: 10.1093/ije/dyn041
85. Orwoll E, Blank JB, Barrett-Connor E, et al. Design and baseline characteristics of the osteoporotic fractures in men (MrOS) study — A large observational study of the determinants of fracture in older men. *Contemp Clin Trials* 2005;26:569-85. doi: 10.1016/j.cct.2005.05.006
86. The ARIC Investigators. The Atherosclerosis Risk In Communities (ARIC) study: design and objectives. *Am J Epidemiol* 1989;129:687-702.

For peer review only

For peer review only - http://bmjopen.bmj.com/site/about/guidelines.xhtml

TABLES

Table 1. Variables planned to be included in IPD meta-analysis modelling.¶

Type	Analysis 1	Analysis 2	Analysis 3	Analysis 4
**Outcomes / DV**	Androgen concentration§	Incident CVD*	Incident deaths (cancer*)	Incident dementia
		Incident deaths (CVD*)	Incident cancer* (diagnoses)	Baseline cognition
		Incident deaths (all-cause)		Change in cognition
**Focal predictor**	-	Androgen concentration§	Androgen concentration§	Androgen concentration§
**Covariates / IV**				
**Demographic**	age	age	age	age
	education level	education level	education level	education level
	ethnicity	ethnicity	ethnicity	ethnicity
	marital status	marital status	marital status	marital status
	site	site	site	site
**Risk factors and comorbidities**	alcohol consumption	alcohol consumption	alcohol consumption	alcohol consumption
	BMI, waist	BMI, waist	BMI, waist	BMI, waist
	physical activity	physical activity	physical activity	physical activity
	smoking status	smoking status	smoking status	smoking status
	BP, hypertension	BP, hypertension	BP, hypertension	BP, hypertension
	general health	general health	general health	general health
		atrial fibrillation		
	prevalent CVD	prevalent CVD		
	prevalent cancer*		prevalent cancer*	
	prevalent dementia			prevalent dementia
	baseline cognition			
	COPD	COPD		
	diabetes	diabetes	diabetes	diabetes
	cholesterol, LDL, HDL	cholesterol, LDL, HDL		
	creatinine level	creatinine level		
	lipid lowering medications	lipid lowering medications		
	anxiety			anxiety
	depression			depression
	psychotropic drug use			psychotropic drug use

Abbreviations: BMI = body mass index; BP= blood pressure; CVD = Cardiovascular Disease; DV = dependent variable; COPD = Chronic Obstructive Pulmonary Disease; IV = independent variables.
¶ = Black font: highly desirable; Green font: only if available.

For peer review only - http://bmjopen.bmj.com/site/about/guidelines.xhtml

737 § = Androgens: total testosterone, dihydrotestosterone, estradiol, luteinising hormone, sex hormone binding globulin.

739 * = Subgroup analyses are also planned. For CVD outcomes: Heart Failure, Myocardial Infarction, Stroke. For cancer outcomes: colorectal cancer, lung cancer, prostate cancer.

For peer review only

For peer review only - http://bmjopen.bmj.com/site/about/guidelines.xhtml

Supplementary Table S1. Example search strategy for systematic review: AIMS study.

The following is the search strategy for MEDLINE. The search strategy for EMBASE, OpenGrey, and Mednar attempts to replicate this as closely as possible, and will be subsequently reported upon completion of the Systematic Review.

1. Testosterone/ or Androgens/
2. (testosterone or androgen* or sex hormone* or sex steroid*).ti.
3. (testosterone or androgen*).ab.
4. cardiovascular diseases/ or heart diseases/ or heart failure/ or vascular diseases/ or stroke/ or myocardial infarction/ or coronary disease/ or cerebrovascular disorders/
5. (cardiovascular or stroke or myocardial infarction or heart failure).ti.
6. neoplasms/ or colorectal neoplasms/ or lung neoplasms/ or prostatic neoplasms/
7. cancer.ti.
8. mortality/ or mortality.ti.
9. dementia/ or cognition/ or dementia.ti. or cognit*.ti.
10. Aging/psychology or Neuropsychological Tests/
11. 1 or 2 or 3
12. 4 or 5 or 6 or 7 or 8 or 9 or 10
13. 11 and 12
14. longitudinal studies/ or prospective studies/ or follow-up studies/ or cohort studies/
15. (prospective or follow-up or cohort study or longitudinal study).ti,ab.
16. 14 or 15
17. 13 and 16
18. (exogenous or replacement or therapy or hormone treatment).ti.
19. Hormone Replacement Therapy/
20. 18 or 19
21. 17 not 20
22. limit 21 to humans
23. limit 22 to english language
24. limit 23 to (adaptive clinical trial or address or autobiography or bibliography or biography or case reports or clinical trial, all or clinical trial, phase i or clinical trial, phase ii or clinical trial, phase iii or clinical trial, phase iv or clinical trial, veterinary or clinical trials, veterinary as topic or clinical trial protocol or clinical trial or comment or controlled clinical trial or dictionary or editorial or lecture or legislation or meta analysis or practice guideline or pragmatic clinical trial or published erratum or randomized controlled trial or retracted publication or "retraction of publication" or "review" or "scientific integrity review" or "systematic review")
25. Retrospective Studies/ or Case-Control Studies/ or (retrospective analysis or case-control).ti.
26. 24 or 25
27. 23 not 26

Notes:

Terms with a trailing “/” are MeSH terms and those with a trailing “*” are truncated search strings. This search strategy is included with the PROSPERO registration for the AIMS

For peer review only

project (Registration No. CRD42019139668). Beforehand, a search of PROSPERO was conducted for another suitable strategy but none were found. However, the above strategy is based upon one that has been used for a similar study.[1]

References cited

1. Holmegard HN, Nordestgaard BG, Jensen GB, Tybjaerg-Hansen A, Benn M. Sex hormones and ischemic stroke: a prospective cohort study and meta-analyses. J Clin Endocrinol Metab. 2016;101:69-78.

For peer review only

PRISMA-IPD Checklist of items to include when reporting a systematic review and meta-analysis of individual participant data (IPD): the Androgens In Men Study (AIMS).

PRISMA-IPD Section/topic	Item No	Checklist item	Reported on page
**Title**			
Title	1	Identify the report as a systematic review and meta-analysis of individual participant data.	1
**Abstract**			
Structured summary	2	Provide a structured summary including as applicable:	4-5
		**Background**: state research question and main objectives, with information on participants, interventions, comparators and outcomes.	
		**Methods**: report eligibility criteria; data sources including dates of last bibliographic search or elicitation, noting that IPD were sought; methods of assessing risk of bias.	
		**Results**: provide number and type of studies and participants identified and number (%) obtained; summary effect estimates for main outcomes (benefits and harms) with confidence intervals and measures of statistical heterogeneity. Describe the direction and size of summary effects in terms meaningful to those who would put findings into practice.	
		**Discussion:** state main strengths and limitations of the evidence, general interpretation of the results and any important implications.	
		**Other:** report primary funding source, registration number and registry name for the systematic review and IPD meta-analysis.	
**Introduction**			
Rationale	3	Describe the rationale for the review in the context of what is already known.	6-7
Objectives	4	Provide an explicit statement of the questions being addressed with reference, as applicable, to participants, interventions, comparisons, outcomes and study design (PICOS). Include any hypotheses that relate to particular types of participant-level subgroups.	8
**Methods**			
Protocol and registration	5	Indicate if a protocol exists and where it can be accessed. If available, provide registration information including registration number and registry name. Provide publication details, if applicable.	5
Eligibility criteria	6	Specify inclusion and exclusion criteria including those relating to participants, interventions, comparisons, outcomes, study design and characteristics (e.g. years when conducted, required minimum follow-up). Note whether these were applied at the study or individual level i.e. whether eligible participants were included (and ineligible participants excluded) from a study that included a wider population than specified by the review inclusion criteria. The rationale for criteria should be stated.	9

For peer review only - http://bmjopen.bmj.com/site/about/guidelines.xhtml

PRISMA-IPD Section/topic	Item No	Checklist item	Reported on page
**Title**			
Identifying studies - information sources	7	Describe all methods of identifying published and unpublished studies including, as applicable: which bibliographic databases were searched with dates of coverage; details of any hand searching including of conference proceedings; use of study registers and agency or company databases; contact with the original research team and experts in the field; open adverts and surveys. Give the date of last search or elicitation.	9
Identifying studies - search	8	Present the full electronic search strategy for at least one database, including any limits used, such that it could be repeated.	9, Suppl. Data
Study selection processes	9	State the process for determining which studies were eligible for inclusion.	9
Data collection processes	10	Describe how IPD were requested, collected and managed, including any processes for querying and confirming data with investigators. If IPD were not sought from any eligible study, the reason for this should be stated (for each such study).<br>If applicable, describe how any studies for which IPD were not available were dealt with. This should include whether, how and what aggregate data were sought or extracted from study reports and publications (such as extracting data independently in duplicate) and any processes for obtaining and confirming these data with investigators.	10-12
Data items	11	Describe how the information and variables to be collected were chosen. List and define all study level and participant level data that were sought, including baseline and follow-up information. If applicable, describe methods of standardising or translating variables within the IPD datasets to ensure common scales or measurements across studies.	12-13
IPD integrity	A1	Describe what aspects of IPD were subject to data checking (such as sequence generation, data consistency and completeness, baseline imbalance) and how this was done.	10-11
Risk of bias assessment in individual studies.	12	Describe methods used to assess risk of bias in the individual studies and whether this was applied separately for each outcome. If applicable, describe how findings of IPD checking were used to inform the assessment. Report if and how risk of bias assessment was used in any data synthesis.	10-11,16-17
Specification of outcomes and effect measures	13	State all treatment comparisons of interests. State all outcomes addressed and define them in detail. State whether they were pre-specified for the review and, if applicable, whether they were primary/main or secondary/additional outcomes. Give the principal measures of effect (such as risk ratio, hazard ratio, difference in means) used for each outcome.	13-16
Synthesis methods	14	Describe the meta-analysis methods used to synthesise IPD. Specify any statistical methods and models used. Issues should include (but are not restricted to):<br>• Use of a one-stage or two-stage approach.<br>• How effect estimates were generated separately within each study and combined across studies (where applicable).	13-16

For peer review only - http://bmjopen.bmj.com/site/about/guidelines.xhtml

PRISMA-IPD Section/topic	Item No	Checklist item	Reported on page
**Title**			
		• Specification of one-stage models (where applicable) including how clustering of patients within studies was accounted for.<br>• Use of fixed or random effects models and any other model assumptions, such as proportional hazards.<br>• How (summary) survival curves were generated (where applicable).<br>• Methods for quantifying statistical heterogeneity (such as I^2 and τ^2).<br>• How studies providing IPD and not providing IPD were analysed together (where applicable).<br>• How missing data within the IPD were dealt with (where applicable).	
Exploration of variation in effects	A2	If applicable, describe any methods used to explore variation in effects by study or participant level characteristics (such as estimation of interactions between effect and covariates). State all participant-level characteristics that were analysed as potential effect modifiers, and whether these were pre-specified.	13-16
Risk of bias across studies	15	Specify any assessment of risk of bias relating to the accumulated body of evidence, including any pertaining to not obtaining IPD for particular studies, outcomes or other variables.	10, 16
Additional analyses	16	Describe methods of any additional analyses, including sensitivity analyses. State which of these were pre-specified.	8, 16-17
**Results**			
Study selection and IPD obtained	17	Give numbers of studies screened, assessed for eligibility, and included in the systematic review with reasons for exclusions at each stage. Indicate the number of studies and participants for which IPD were sought and for which IPD were obtained. For those studies where IPD were not available, give the numbers of studies and participants for which aggregate data were available. Report reasons for non-availability of IPD. Include a flow diagram.	TBA
Study characteristics	18	For each study, present information on key study and participant characteristics (such as description of interventions, numbers of participants, demographic data, unavailability of outcomes, funding source, and if applicable duration of follow-up). Provide (main) citations for each study. Where applicable, also report similar study characteristics for any studies not providing IPD.	TBA
IPD integrity	A3	Report any important issues identified in checking IPD or state that there were none.	TBA
Risk of bias within studies	19	Present data on risk of bias assessments. If applicable, describe whether data checking led to the up-weighting or down-weighting of these assessments. Consider how any potential bias impacts on the robustness of meta-analysis conclusions.	TBA
Results of individual studies	20	For each comparison and for each main outcome (benefit or harm), for each individual study report the number of eligible participants for which data were obtained and show simple summary data for each intervention group (including, where applicable, the number of events), effect estimates and confidence intervals. These may be tabulated or included on a forest	TBA

For peer review only - http://bmjopen.bmj.com/site/about/guidelines.xhtml

PRISMA-IPD Section/topic	Item No	Checklist item	Reported on page
**Title**			
		plot.	
Results of syntheses	21	Present summary effects for each meta-analysis undertaken, including confidence intervals and measures of statistical heterogeneity. State whether the analysis was pre-specified, and report the numbers of studies and participants and, where applicable, the number of events on which it is based.	TBA
		When exploring variation in effects due to patient or study characteristics, present summary interaction estimates for each characteristic examined, including confidence intervals and measures of statistical heterogeneity. State whether the analysis was pre-specified. State whether any interaction is consistent across trials.	
		Provide a description of the direction and size of effect in terms meaningful to those who would put findings into practice.	
Risk of bias across studies	22	Present results of any assessment of risk of bias relating to the accumulated body of evidence, including any pertaining to the availability and representativeness of available studies, outcomes or other variables.	TBA
Additional analyses	23	Give results of any additional analyses (e.g. sensitivity analyses). If applicable, this should also include any analyses that incorporate aggregate data for studies that do not have IPD. If applicable, summarise the main meta-analysis results following the inclusion or exclusion of studies for which IPD were not available.	TBA
**Discussion**			
Summary of evidence	24	Summarise the main findings, including the strength of evidence for each main outcome.	TBA
Strengths and limitations	25	Discuss any important strengths and limitations of the evidence including the benefits of access to IPD and any limitations arising from IPD that were not available.	TBA
Conclusions	26	Provide a general interpretation of the findings in the context of other evidence.	TBA
Implications	A4	Consider relevance to key groups (such as policy makers, service providers and service users). Consider implications for future research.	TBA
**Funding**			
Funding	27	Describe sources of funding and other support (such as supply of IPD), and the role in the systematic review of those providing such support.	20-21

For peer review only - http://bmjopen.bmj.com/site/about/guidelines.xhtml

A1 – A3 denote new items that are additional to standard PRISMA items. A4 has been created as a result of re-arranging content of the standard PRISMA statement to suit the way that systematic review IPD meta-analyses are reported.

© Reproduced with permission of the PRISMA IPD Group, which encourages sharing and reuse for non-commercial purposes

For peer review only

For peer review only - http://bmjopen.bmj.com/site/about/guidelines.xhtml

For peer review only

For peer review only - http://bmjopen.bmj.com/site/about/guidelines.xhtml
